# APR-246 as a radiosensitization strategy for mutant p53 cancers treated with alpha-particles-based radiotherapy

**DOI:** 10.1038/s41419-024-06830-3

**Published:** 2024-06-18

**Authors:** Or Michaeli, Ishai Luz, Maayan Vatarescu, Tal Manko, Noam Weizman, Yevgeniya Korotinsky, Alexandra Tsitrina, Alex Braiman, Lior Arazi, Tomer Cooks

**Affiliations:** 1 The Shraga Segal Department of Microbiology, Immunology & Genetics, Faculty of Health Sciences, Ben-Gurion University, Beer-Sheva, Israel; 2 Translational Research Laboratory, Alpha Tau Medical, Jerusalem, Israel; 3 Unit of Nuclear Engineering, Faculty of Engineering Sciences, Ben-Gurion University of the Negev, Beer-Sheva, Israel; 4 Ilse Katz Institute for Nanoscale Science and Technology, Ben-Gurion University of the Negev, Beer Sheva, Israel

**Keywords:** Radiotherapy, Targeted therapies

## Abstract

Radiation therapy (RT) remains a common treatment for cancer patients worldwide, despite the development of targeted biological compounds and immunotherapeutic drugs. The challenge in RT lies in delivering a lethal dose to the cancerous site while sparing the surrounding healthy tissues. Low linear energy transfer (low-LET) and high linear energy transfer (high-LET) radiations have distinct effects on cells. High-LET radiation, such as alpha particles, induces clustered DNA double-strand breaks (DSBs), potentially inducing cell death more effectively. However, due to limited range, alpha-particle therapies have been restricted. In human cancer, mutations in TP53 (encoding for the p53 tumor suppressor) are the most common genetic alteration. It was previously reported that cells carrying wild-type (WT) p53 exhibit accelerated senescence and significant rates of apoptosis in response to RT, whereas cells harboring mutant p53 (mutp53) do not. This study investigated the combination of the alpha-emitting atoms RT based on internal Radium-224 (^224^Ra) sources and systemic APR-246 (a p53 reactivating compound) to treat tumors with mutant p53. Cellular models of colorectal cancer (CRC) or pancreatic ductal adenocarcinoma (PDAC) harboring mutant p53, were exposed to alpha particles, and tumor xenografts with mutant p53 were treated using ^224^Ra source and APR-246. Effects on cell survival and tumor growth, were assessed. The spread of alpha emitters in tumors was also evaluated as well as the spatial distribution of apoptosis within the treated tumors. We show that mutant p53 cancer cells exhibit radio-sensitivity to alpha particles in vitro and to alpha-particles-based RT in vivo. APR-246 treatment enhanced sensitivity to alpha radiation, leading to reduced tumor growth and increased rates of tumor eradication. Combining alpha-particles-based RT with p53 restoration via APR-246 triggered cell death, resulting in improved therapeutic outcomes. Further preclinical and clinical studies are needed to provide a promising approach for improving treatment outcomes in patients with mutant p53 tumors.

## Introduction

Alongside many other commonly used anti-cancer strategies and despite recently developed modalities that include targeted biological compounds and immunotherapeutic drugs, most cancer patients worldwide are still treated with some form of radiation therapy (RT), either as a main treatment or as an adjuvant approach [1–3]. The major challenge in RT lies in the delicate balance between delivering a destructive, lethal dose into the cancerous tissue while limiting the damage to the surrounding healthy perimeters and the radiotoxicity affecting vital organs. To that end, the use of low versus high linear energy transfer (low-LET and high-LET) differs significantly [4–6]. While 1 Gray (Gy) dose of X-rays (low-LET) induces thousands of single-strand breaks (SSBs) to the DNA of a mammalian cell and approximately 40 double strand breaks (DSBs), as LET increases, so does cytotoxicity [7, 8]. Notably, only a few hits of alpha particles (high-LET) to the DNA can produce clustered DSBs that will drive the cell towards an apoptotic course, mainly due to their high ionization density. Clustered DSBs are repaired less efficiently than isolated damage, suggesting that high-LET radiation therapy should be more robustly used for the eradication of tumors [9, 10].

Nevertheless, the vast majority of cancer patients are subjected to low-LET (most often X-rays) RT due to the heavy mass of alpha particles leading to strong interactions with tissue and their slow movement [11, 12]. Hence, the limited range of alpha particles in tissues (less than 100μm) has traditionally been considered a limiting factor, restricting the development of alpha-particle-based therapies [13].

Radiosensitizing agents (radiosensitizers) are substances and compounds which have the capacity to augment the effect produced by RT alone [14–17]. While some radiosensitizers work as oxygen mimicking molecules or by suppressing radioprotective mechanisms in the cell, there are also agents which enhance apoptosis by activating DNA damage repair factors, such as p53 [18–20].

The wild-type (WT) form of the transcription factor p53 is known to enhance genomic stability and hinder tumorigenesis by promoting cell cycle arrest, allowing damaged DNA to be repaired preceding DNA synthesis [21–23]. Moreover, specific transcriptional profiles induced by p53, can permanently eliminate damaged and potentially mutated cells from the dividing cell population [24]. Notably, following DNA-damage inducing therapies such as RT, p53 is a key molecule in mediating cancer cell radio-sensitivity [18]. It was previously reported that cells carrying WT p53 exhibit accelerated senescence and significant rates of apoptosis in response to RT, whereas cells harboring mutant p53 (mutp53) do not [25–27]. Furthermore, transgenic mice carrying mutp53 were observed with increased resistance to γ-irradiation and that overexpression of specific mutants increased radiation resistance of mouse hematopoiesis significantly. Notably, different mutant sites of p53 are differentially sensitive to radiotherapy [28, 29].

Since most ‘hotspot’ p53 mutants are a result of a point missense mutation in the TP53 gene, the high similarity between the WT and those mutants have led to multiple efforts attempting to slow tumor progression by restoring the WT activity of mutp53 [30, 31]. Ample evidence has accumulated suggesting that small molecule compounds and peptides may change the folding pattern of mutp53 and restore the WT structure, some of these compounds are currently undergoing clinical trials [32–35]. APR-246 (also known as PRIMA-1MET), is an example of a compound that can restore the WT p53 conformation and anti-tumor transcriptional activity by covalently binding the DNA binding domain of mutp53 [36–38]. It has shown significant anti-tumor activity in various cancers, including esophageal adenocarcinoma, acute myeloid leukemia, and triple negative breast cancer [39–41]. In the context of low-LET RT, a recent study indicated that APR-246 is mediating radiosensitization effects through both p53-dependent as well as p53-independent manners [42].

In this study, we combined an alpha-particle-based RT with APR-246 for the treatment of tumors harboring mutp53. We used the RT to provoke irreparable DNA-damage then harnessed p53-restoring strategy to induce apoptosis in cells that were not destroyed by the RT itself. We show that in cancer models endogenously carrying ‘hotspot’ mutations in p53, combining alpha-particle-based RT with p53 reactivation have the potential to promote primary tumor control and yield a beneficial treatment response.

## Results

### HCT116 cells harboring mutant p53 show radiosensitivity when exposed to alpha particles

To investigate the radio-sensitivity of cancer cells based on their p53 status, we used the HCT116 cellular model, typically not harboring the gain-of-function p53 mutant. We used the isogenic set of cells comparing the mutant p53 in the 248 position (arginine to tryptophan, R248W), the WT (p53 +/+) and the Null p53 cells (p53 −/−) described in [43, 44].

**Fig. 1:**
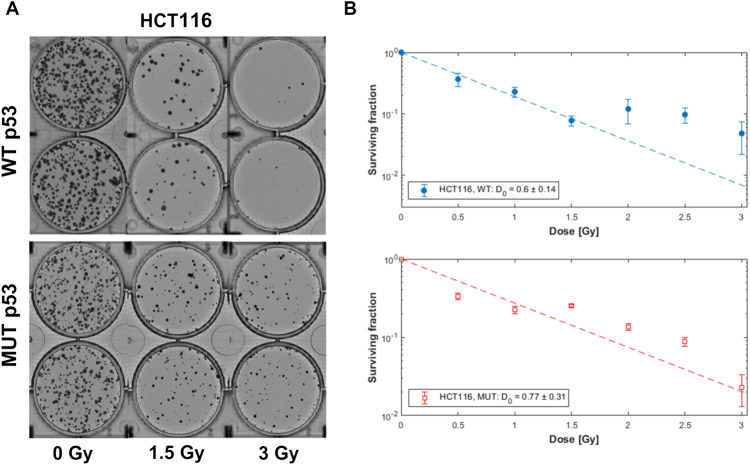
HCT116 cells harboring mutant p53 show radiosensitivity when exposed to alpha particles. **A** Colony formation assay of HCT116 cells exposed to alpha particles. **B** Exposure of HCT116 cells differing by their p53 status (WT, Mutant) to different doses of alpha particle fluxes (0–3 Gy in 0.5 Gy intervals). D0 represent the dose needed to be deposited in order to reduce colony formation to 37% of the unirradiated control.

HCT116 cells differing by their TP53 status, were subjected to increasing doses of α irradiation, in an Americium-241(^241^Am) irradiation station as described in [45] as well as in the methods section. Notably, while the obtained survival curves show that α-irradiation is more damaging for the cells with WT p53 status, the cells harboring mutated p53 were also significantly affected (Fig. 1A, B). We observed that when cells were irradiated with 1.5 Gy of alpha particles, 25.2% of the cells with the mutant p53 survived compared to 7.8% in the WT p53 cells. Altogether, the D0 parameter (indicating the radiation energy necessary to limit survival of colonies to 37% of the unirradiated control) was slightly higher in the mutap53 cells (0.77) compared to the WT cells (0.6). Notably, in the 2 and 2.5 Gy treatments, no significant difference was observed in the survival between the WT and MUT colonies, suggesting that p53-independent mechanisms are more dominant when cells are exposed to higher doses of alpha particle fluxes. The radio-sensitivity of the mutp53 cells to alpha particles led us to harness additional therapy in order to obtain higher sensitivity at low radiation levels. Altogether, and unlike previous observations made when low-LET RT was used [27, 46]the colony formation assay indicated that alpha particles significantly affected the survival of HCT116 cells harboring both WT or mutant p53.

**HCT116 xenografts carrying the R248W mutant p53 are sensitive to alpha-particle-based RT** To corroborate the observation that mutant p53 tumor cells are susceptible when exposed to alpha particles also in vivo, we used intratumoral insertion of sources coated with Radium-224 (^224^Ra). These ^224^Ra-coated sources employ dispersed alpha particles, shed by emitting atoms of the ^224^Ra decay chain (Supplementary Fig. 1), with the goal to eradicate malignant tissues [47–50]. The decay of ^224^Ra, which have 3.7 days half-life, releases short-lived daughter radionuclides that disperse in the tumor and deposit highly destructive alpha particles into the tumor [51, 52].

**Fig. 2:**
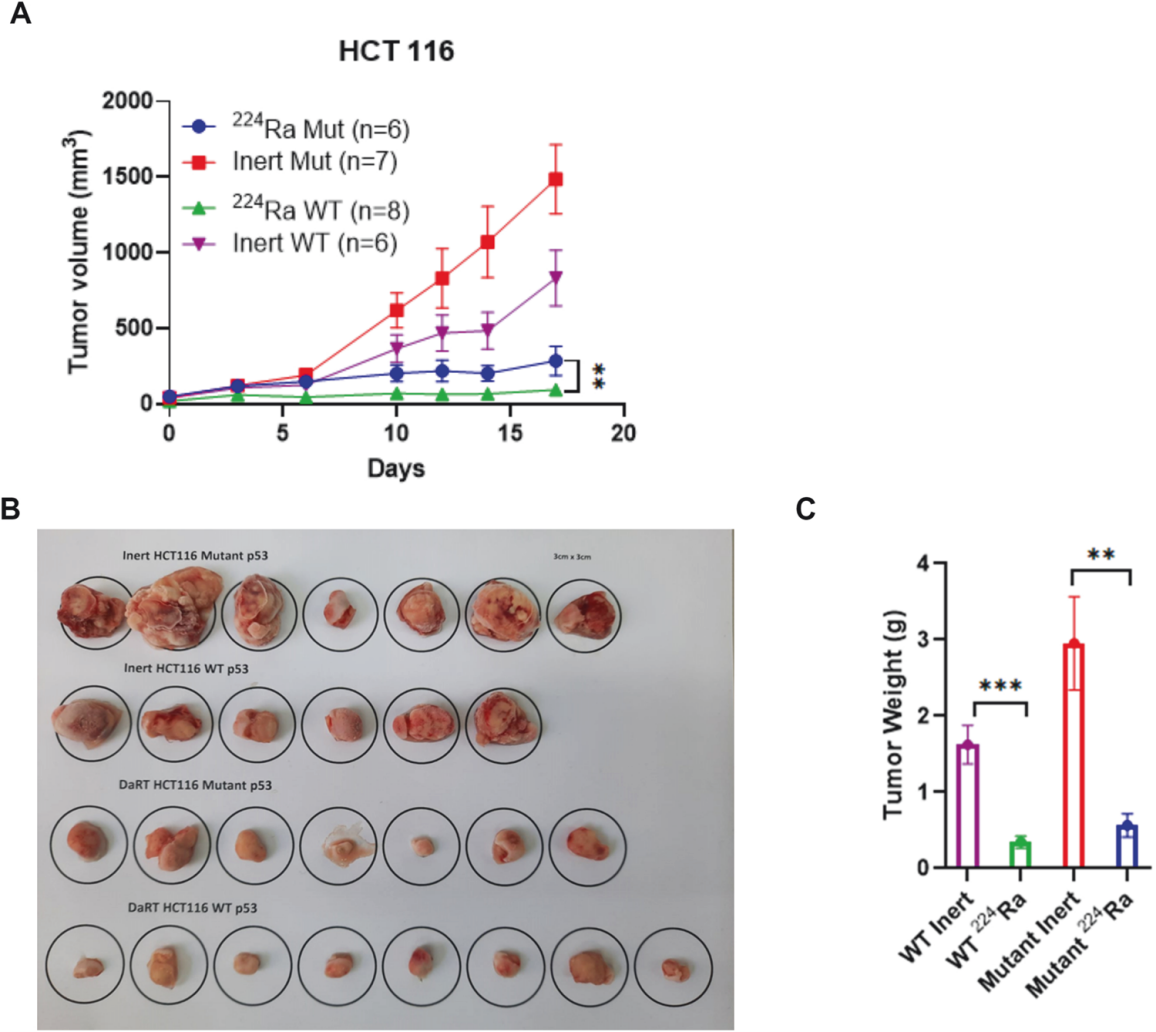
Tumors carrying GOF p53 mutants are more resistant to high-LET RT. HCT116 tumors differing by p53 status (WT and MUT) were transplanted as xenografts, treated with either Inert or ^224^Ra sources and monitored for tumor growth and weight) **A** Tumor volumes ± SEM, (**P-value of 0.01). **B** At the endpoint (day 17), tumors were removed and photographed. **C** Tumor were weighed at the endpoint of the experiment (day 17). (**P-value of 0.01), (***P-value = 0.001).

We transplanted HCT116 xenografts (either mutant or WT p53) on the back of immune suppressed (nude) mice. When tumors reached an average volume of 40–50 mm^3^ we treated them with a single ^224^Ra source and monitored tumor growth compared to tumor-bearing mice treated with non-radioactive source (Inert). The tumor growth rate in mice bearing the mutant p53 tumors was significantly faster. After 17 days from tumor inoculation, the average volume of the inert-treated HCT116 Mutant tumors was 1485 mm^3^ compared to 832 mm^3^ in the inert-treated WT tumors. Nevertheless, in the ^224^Ra treated groups, the HCT116 mutant tumors were robustly affected by the alpha-particle-based RT which led to a fivefold decrease in average volume. We therefore concluded that both WT and mutant p53 HCT116 tumors were significantly sensitive to the RT treatment as can be seen in Fig. 2A and Supplementary Fig. 2. After the last volume measurement at day 17 post treatment, the tumors were also removed and weighted. The average weight of the ^224^Ra-treated WT HCT116 tumors was 0.347 g, while the ^224^Ra-treated mutp53 tumors weighted 0.565 g in average (Fig. 2B, C).

### Alpha radiation combined with p53 reactivation yielded an increase in radio-sensitivity

To investigate the radio-sensitivity of cancer cells based on their p53 status, we used additional two cellular models: PANC-1 cells (pancreatic ductal adenocarcinoma, PDAC) and HT-29 cells (colorectal cancer, CRC). For the PANC-1, endogenously harboring the gain-of-function (GOF) p53 mutant in the 273 position (arginine to histidine, R273H), we used negative control cells where the mutant p53 was knocked out (KO) using CRISPR/Cas9 as described in [53, 54]. The HT-29 cells, endogenously carry a TP53 mutation in the 273 position (arginine to histidine, R273H) and we also used a stable mutant p53 ‘knock-down’ strategy (shRNA, as detailed in the ”Methods” section).

**Fig. 3:**
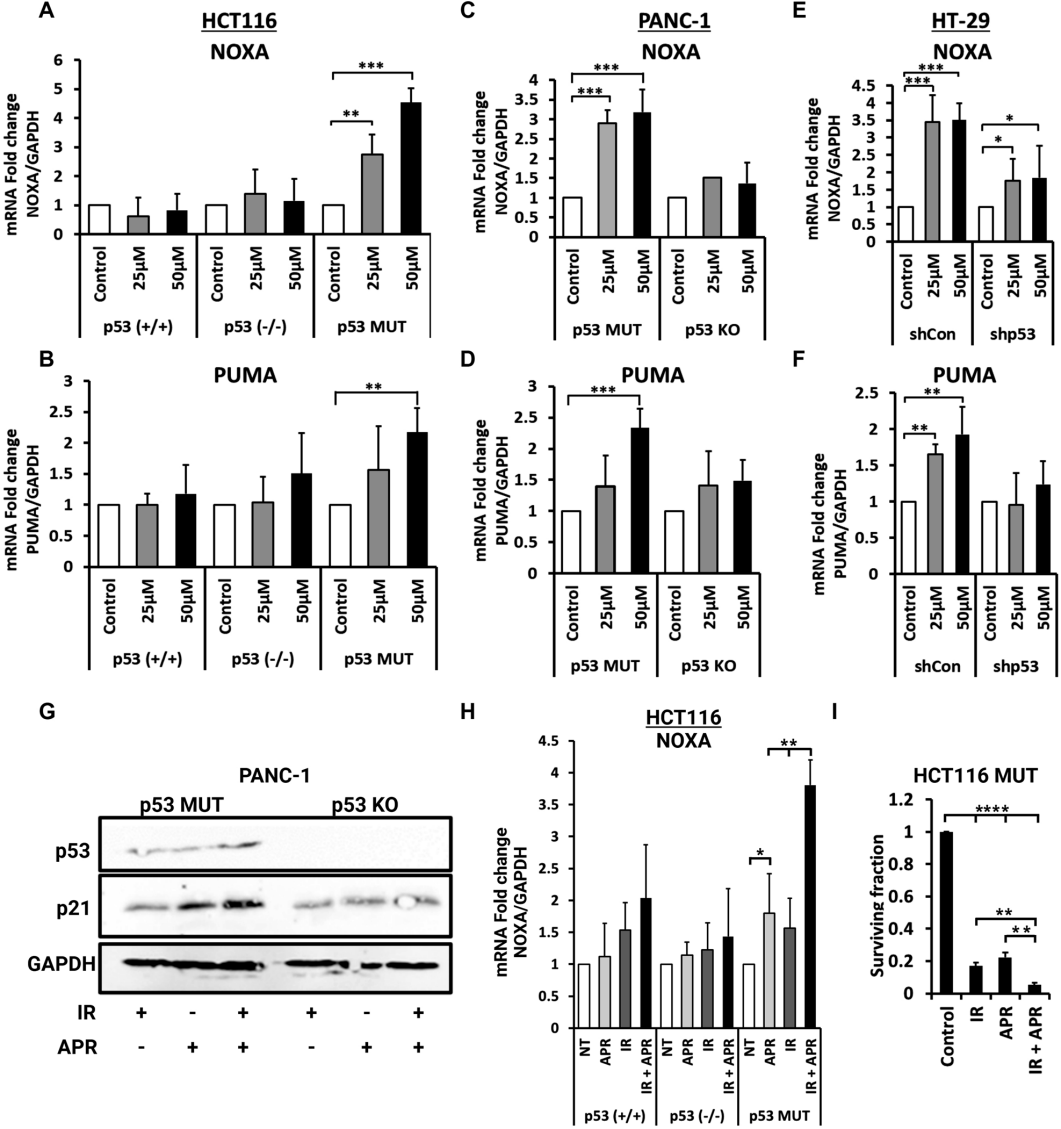
Alpha radiation combined with p53 reactivation yielded an increase in radio-sensitivity. HCT-116, PANC-1 and HT-29 cells were treated with APR-246 for 8 h using 2 concentrations compared with untreated control (0, 25 and 50 µM). RNA was extracted and subjected to qRT-PCR analysis with primers specific for NOXA and PUMA mRNA. Values were normalized for GAPDH mRNA in the same sample and presented as fold change relative to the control cells not treated with APR-246. **A** NOXA levels in HCT-116 cells differing by their p53 status (p53 +/+, p53 -/- and p53 MUT). **B** PUMA levels in HCT-116 cells differing by their p53 status (p53 +/+, p53 -/- and p53 MUT). **C** NOXA levels in PANC-1 cells differing by their p53 status (p53 MUT and p53 KO). **D** PUMA levels in PANC-1 cells differing by their p53 status (p53 MUT and p53 KO). **E** NOXA levels in HT-29 cells differing by their p53 status (shCon and shp53). **F** PUMA levels in HT-29 cells differing by their p53 status (shCon and shp53). **G** Mutp53 and p21 expression levels in PANC-1 cells irradiated by 1 Gy of X-rays, treated with APR-246 (75 µM) or both. GAPDH was used as a loading control. **H** HCT-116, cells were irradiated with alpha particles (1 Gy) and then treated with APR-246 (25 µM) for additional 8 h and compared with untreated cells or cells treated with either IR or APR-246. RNA was extracted and subjected to qRT-PCR analysis with primers specific for NOXA mRNA. Values were normalized for GAPDH mRNA in the same sample and presented as fold change relative to the control cells not treated with APR-246. **I** Colony formation assay of HCT116 mutp53 cells treated with 1 Gy of alpha irradiation (IR), 25 µM of APR-246 (APR-246) or a combination (IR + APR-246), compared to a non-treated control.

Since alpha particles produce high levels of DNA damage and since the cellular response to the damage depends on the status of p53, we hypothesized that augmenting the p53 response in alpha-irradiated cells will further increase their radio-sensitivity, capitalizing on the activation of transcriptional programs governed by p53. We chose APR-246 as a small compound known to restore the WT conformation in various p53 mutants, therefore reactivating p53 as a transcription factor protecting DNA integrity. First, we wanted to corroborate that APR-246 activates the mutant p53 as reported in previous studies [55]. To that end, we treated cancer cells harboring GOF p53 mutants with APR-246 to verify that p53 targets are upregulated. As can be seen in Fig. 3A–F, treatment with 25 or 50 μM of APR-246 resulted in a significant upregulation of both proapoptotic p53 target genes ‘p53 Up-Regulated Modulator Of Apoptosis’ (PUMA) and ‘Phorbol-12-myristate-13-acetate-induced protein 1’ (PMAIP1, NOXA) when the cells carried mutant p53. NOXA and PUMA levels were significantly elevated in HCT116 MUT but not in the p53 WT or NULL derivates (Fig. 3A, B). This APR-246-induced-upregulation of both proapoptotic genes was noticeable also in PANC-1 cells but not when the mutant p53 was knocked out (Fig. 3C, D). In addition, both NOXA and PUMA RNA levels were overexpressed in HT-29 cells exposed to APR-246. This overexpression was less significant in the HT-29 shp53 cells (Fig. 3E, F). On the protein level, we induced DNA damage (using X-rays, 1 Gy) in PANC-1 cells harboring mutp53 and followed the DNA damage with an APR-246 treatment. We compared the mutp53 PANC-1 cells to PANC-1 cells where mutp53 was knocked out (KO). As expected, no p53 was detected in the KO cells and APR-246 did not affect the expression levels of p53 targets represented by CDKN1A (p21). In the PANC-1 cells where endogenous mutp53 is present, a clear activation of p21 was observed with the APR-246 treatment after DNA-damage was inflicted (Fig. 3G). After establishing that APR-246 is increasing p53 activity, we continued to test the combined effect of alpha particles with APR-246 on cancer cells harboring mutp53. HCT116 MUT cells were irradiated with alpha particles (1 Gy) and then added with 25 μM of APR-246 for 8 h. NOXA expression levels were shown (in Fig. 3G) to significantly increase compared with each treatment alone (either the irradiation of the drug). We also observed an increase in NOXA and cleaved-Caspase3 levels in PANC-1 and HT-29 cells treated with both IR and APR (Supplementary Fig. 3A). We also conducted colony formation assays on HCT116 mutp53 treated with 1 Gy of alpha irradiation (IR) alone, APR-246 alone (APR) as well as a combination of IR and the drug (IR + APR). As seen in Supplementary Fig. 3B and quantified in Fig. 3I, while each monotherapy affected the surviving fraction of colonies, the combined treatment yielded a robust effect on cell survival compared to each of the treatments separately. The combined treatment of IR and APR also promoted a significant increase in cell death in PANC-1 cells (Supplementary Fig. 3C).

### Combination of high-LET RT with APR-246 attenuated tumor growth of CRC and PDAC xenografts compared to either monotherapy

Based on our in vitro observations we wanted to test in vivo, whether mutant p53 tumors will be more susceptible to a combination therapy of our alpha-particle-based technique (internal ^224^Ra sources) and APR-246.

**Fig. 4:**
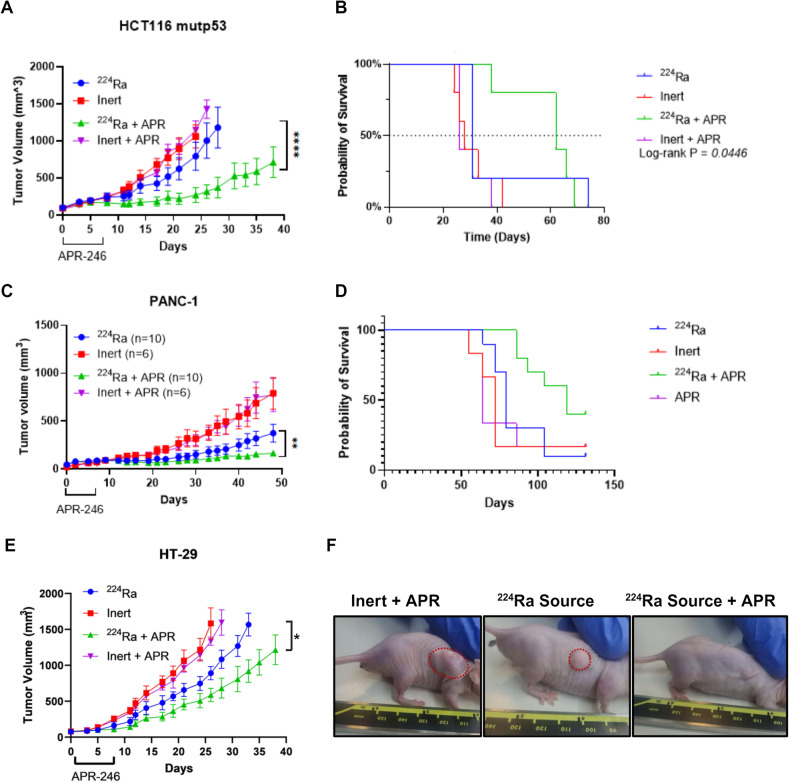
Combination of high-LET ^224^Ra source with APR-246 attenuated tumor growth of CRC and PDAC xenografts compared to either monotherapy. **A** Mean tumor volume ± SEM of HCT116-bearing mice (~90 mm^3^ average volume) were treated with a 75-kBq ^224^Ra source or inert source on day 0, followed by 14 doses of 50 mg/kg APR-246 *i.p* on days 1–7. (***p < 0.005), (*n* = 6 in each treatment group). **B** Kaplan-Meier survival plots for (**A**). **C** Mean tumor volume ± SEM of PANC-1-bearing mice (~45 mm^3^ average volume) were treated with a 75-kBq ^224^Ra source or inert source on day 0, followed by 14 doses of 50 mg/kg APR-246 i.p. on days 1–7. (***p < 0.005). **D** Kaplan-Meier survival plots for (**C**). **E** Mean tumor volume ± SEM of HT-29-bearing mice (~85 mm^3^ average volume) were treated with a 75-kBq ^224^Ra source or inert source on day 0, followed by 14 doses of 50 mg/kg APR-246 i.p. on days 1–7. (*p < 0.05), (*n* = 6 in each treatment group). **F** Representative photos of tumor-bearing mice taken 60 days post treatments.

To determine the effect of ^224^Ra-sources in combination with the systemic administration of APR-246 in vivo, HCT116 mut p53 xenografts were allowed to grow for 15 days to an average volume of ~90 mm^3^. Thereafter, each tumor was treated with either a single ^224^Ra source or inert (non-radioactive control) source. APR-246 or PBS control treatment began 1 day post source insertion for 7 days (50 mg/kg twice a day, morning and evening for a total of 14 doses spanning over 7 days). ^224^Ra (*n* = 5) as standalone treatments provided a significant attenuation in tumor growth compared to the Inert+PBS control (*n* = 5) group (*p* < 0.0021). APR as standalone treatment did not show a significant affect compared to the Inert+PBS control group. ^224^Ra-treated tumors were significantly smaller compared to the APR-treated group (*p* < 0.0017). The most robust effect on tumor growth was observed in the combined therapy group (*n* = 5) compared to APR (*p* < 0.0001), ^224^Ra alone (*p* < 0.0001), or control (*p* < 0.0001) (Fig. 4A). We concluded that in CRC tumors harboring mutp53, the combination of alpha-particles-based RT with a p53 restoring agent, yielded a significantly superior treatment approach with the potential to increase efficacy. The strong effect of ^224^Ra + APR-246 on tumor growth was also manifested in prolonged survival. Mice treated with the combination treatment survived for a significantly longer period in average (*p* = 0.0446) as could be seen in Fig. 4B.

To delineate whether the effect of the combined treatment is associated with p53 status, we repeated the same experiments, with HCT116 tumors harboring either WT p53 (+/+) or lacking p53 (NULL, p53 −/−). Notably, as can be observed in Supplementary Fig. 5A, B, we could not detect any added value for the systemic administration of APR-246 in tumors not harboring mutant p53.

^224^Ra sources and APR-246 combination was also tested in the PDAC model. PANC-1 mutp53 xenografts were transplanted and allowed to grow for 11 days to an average volume of ~40 mm^3^. The tumors were treated similarly to the above-mentioned CRC tumors (APR-246 treatment began 1-day post ^224^Ra using the same dosage). ^224^Ra (*n* = 10) as a monotherapy provided a significant attenuation in tumor growth compared to the Inert+PBS control (*n* = 6) group (*p* < 0.0001). APR as a monotherapy did not show a significant effect compared to the Inert+PBS control group. ^224^Ra-treated tumors were significantly smaller compared to the APR-treated group (*p* < 0.0001). Here, as well, the most robust effect on tumor growth was observed in the combined therapy group (*n* = 10) compared to APR (*p* < 0.0001), ^224^Ra alone (*p* < 0.0001), or control (*p* < 0.0001) (Fig. 4C). The combination of ^224^Ra and APR-246 also yielded prolonged survival in the PANC-1 model, while no difference in life expectancy was observed between the control treated group and each monotherapy (Fig. 4D).

Our findings were corroborated in the HT-29 model as well, as xenografts were injected subcutaneously and allowed to grow to an average volume of ~85 mm^3^. In this model, in-line with the HCT116 and PANC-1 in-vivo experiments, the combination of ^224^Ra and APR-246 was significantly superior and yielded a notable attenuation in tumor growth (Fig. 4E). Figure 4F presents photos of several animals treated with either monotherapy or the combination. Notably, in both HCT116 and PANC-1 models, when we treated larger tumors (200 mm^3^ average volume in the HCT116 tumors and 100 mm^3^ average volume in the PANC-1 tumors), we did not observe a significantly beneficial effect to the combination compared with the ^224^Ra source alone arm (Supplementary Fig. 4A, B). These findings are in accordance with previous preclinical models treated with a single ^224^Ra source [45]. When the treated tumors are too large and the diffusion length of the radioactive atoms will not affect the periphery of the tumors, the effect on tumor growth is limited and often not observed.

### APR-246 treatment did not affect the spread of alpha emitters in the tumor or reduced the clearance of ^212^Pb through the blood

**Fig. 5:**
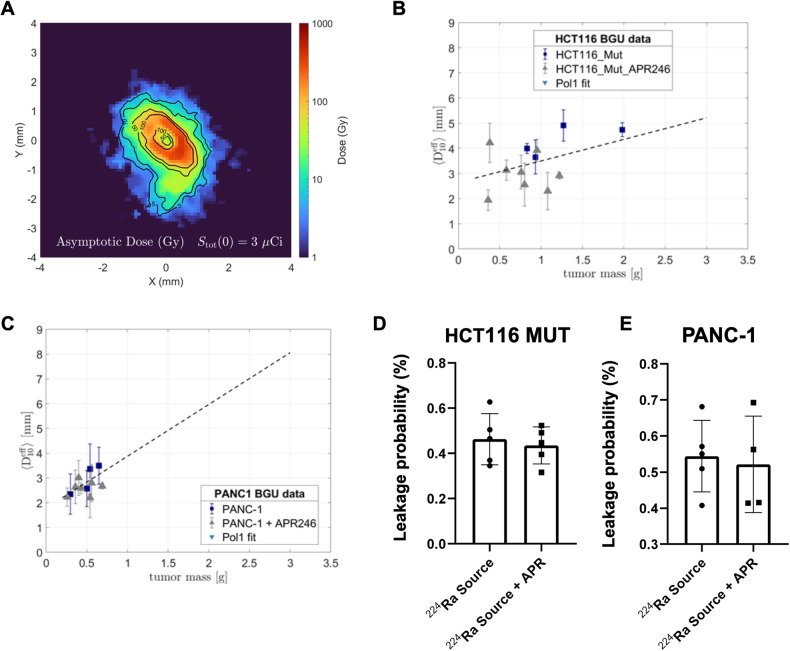
The effect of APR-246 on the spread of alpha emitters in the tumor or the clearance of ^212^Pb. **A** A representative autoradiography image of a HCT116 tumor treated by a single ^224^Ra source and APR-246, in raw photo-stimulated luminescence (PSL) units with the PSL map translated to estimated dose. Effective diameter of the region subject to an estimated macroscopic ^212^Bi/^212^Po alpha dose of >10 Gy as a function of the tumor mass, for HCT116 tumors (**B**) or PANC-1 tumors (**C**) treated by ^224^Ra + APR-246 and by ^224^Ra + PBS as control. The ^212^Pb leakage probability from HCT116 tumors (**D**) or PANC-1 tumors (**E**).

To validate that the systemic administration of APR-246 does not interfere with the diffusion of the alpha-emitting atoms released by the ^224^Ra source, we used autoradiography analysis. As discussed in [56, 57], the physical model predicts that the spread of ^212^Pb (the daughter radionuclide, part of the ^224^Ra decay chain) inside the tumor decreases with increasing rate of its clearance through the blood. Here, we quantified the spread by the effective diameter of the region in which the local measured ^212^Pb activity translated to an estimated macroscopic alpha dose of >10 Gy by the alpha emissions of the decay chain progeny. The clearance rate of ^212^Pb is quantified by its leakage probability from the tumor. To investigate this, autoradiography experiments and measurements of the ^212^Pb leakage probability were performed on HCT116 and PANC-1 tumors, where the tumors were treated with a single ^224^Ra source combined with either APR or PBS as control, following the same treatment regimen employed in the efficacy experiments presented in Fig. 4. Figure 5A shows a representative autoradiography image of a treated tumor. The effective diameter in the case of ^224^Ra + APR did not change significantly compared to ^224^Ra + PBS for the same tumor mass in both examined tumor models (Fig. 5B, C). We also determined that the ^212^Pb leakage probability from the tumors did not differ significantly between the two treatment groups (Fig. 5D). We concluded that when APR-246 is added systemically, no major effects were recorded on the manner by which the alpha-emitting atoms diffused in the tumor tissue and were evacuated out of the tumor.

### APR-246 treatment extends the apoptotic signal when combined with alpha-particle-based RT

**Fig. 6:**
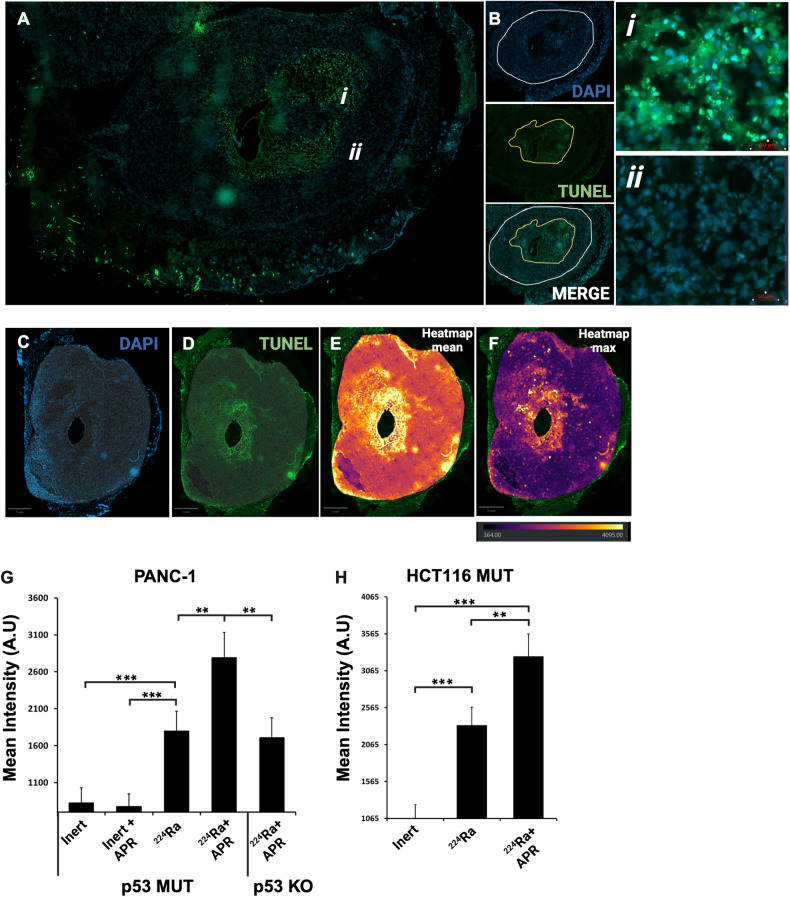
Apoptotic ‘kill-region’ extended in tumors treated with ^224^Ra source and Apr-246. **A** A representative section of an HCT116 mutp53 tumor treated with ^224^Ra source and APR-246 and stained with TUNEL and DAPI. **B** Circled region of the apoptotic signal in green, and the tumor perimeters in white. *i* – represents an area with positive TUNEL staining. *ii*- represents an area with negative TUNEL staining. **C** A representative section of a PANC-1 tumor treated with ^224^Ra source and APR-246 stained with DAPI (**C**) and TUNEL (**D**) and the spatial intensity of the signal was quantified in QuPath software to form a heatmap of the signal recorded in the green channel (TUNEL) using 50 nM tiles surrounding the insertion point of the ^224^Ra source (**E**, **F**). **G** Based on the heatmaps generated, apoptotic signal intensity was quantified for the PANC-1 model in tumors treated with ^224^Ra source + APR-246 and compared with Inert, Inert + APR-246 and ^224^Ra alone. Each ^224^Ra group consisted of 5 tumors while the Inert groups consisted 3 tumors each. In addition, PANC-1 p53 KO tumors were treated and similarly assessed. (**p < 0.05), (***p < 0.01). **H** Same methodology and analysis as in (**G**), was used with HCT116 MUT tumors. Here, ^224^Ra source + APR-246 treatment was compared to Inert and ^224^Ra source alone. Each ^224^Ra group consisted of 5 tumors while the Inert group consisted 3 tumors each. (**p < 0.05), (***p < 0.01).

To corroborate the mechanism by which ^224^Ra source and APR-246 increase efficacy and achieve better tumor control, we measured the spatial distribution of apoptotic cell death in treated tumors. To that end, we treated PDAC and CRC tumors either with ^224^Ra source alone or with ^224^Ra source combined with APR-246 (50 mg/kg per day) and allowed 5 days for the spread of radioactive atoms and the apoptotic signal. After tumors were removed, fixed and sectioned, a Tunel assay was conducted on sections from both treatment groups and the signal was measured and evaluated (Fig. 6A, B). Heatmap analysis of the area and intensity of the apoptotic signal indicated a significant increase in the intensity of apoptosis in tumors treated with both ^224^Ra source and APR-246 compared to tumors treated with the ^224^Ra source alone (Fig. 6C–H). PANC-1 tumors treated with both ^224^Ra source and APR-246 were measured with a significantly intensified apoptotic signal surrounding the source insertion point when compared with other treatments (either Inert, Inert + APR-246 or 224Ra source + APR-246) (Fig. 6G). Moreover, the combined treatment yielded a significant increase in apoptotic signal also when we compared to PANC-1 p53 KO tumors, suggesting the relevance of p53 presence for the effect on tumor growth (Fig. 6G). These findings were corroborated using the HCT116 MUT tumors that were also treated with ^224^Ra source alone and gave rise to a reduced apoptotic signal surrounding the source insertion point compared with the same tumors treated with the combination (Fig. 6H). We therefore concluded that the addition of APR-246 to the alpha-particle-based RT increase apoptosis induced by each ^224^Ra source when inserted into the middle of a mutp53 tumor (Supplementary Fig. 6).

## Discussion

The present study aimed to investigate the impact of p53 status on the response to high LET RT in cancer cells and evaluate the potential of a p53 reactivation strategy to enhance the efficacy when diffusing alpha emitting RT is applied to treated tumors with mutation in TP53. Our findings provide valuable insights into the underlying mechanisms of radio-resistance in tumors with mutant p53 and highlight a promising therapeutic approach with the potential to improve treatment outcomes.

Our results demonstrated that in high-LET, represented in our study by alpha particles, the presence of mutant p53 tumors harboring mutant p53 exhibited a profound sensitivity in vitro as well as to the ^224^Ra treatment in vivo for the PANC-1 model as well as in the HCT-116 model when the treated tumors were small enough. In this study, we show that PANC-1 tumors harboring the GOF R273H mutant p53 were found to be significantly responsive to the ^224^Ra source treatment alone indicating that in certain conditions, when the diffusion of the ^224^Ra daughter atoms can affect the entire tumor perimeters, the presence of mutant p53 should not be a ‘molecular barrier’ to the ability of alpha particles to destruct cancer cells and abolish tumors. This observation is in contrast to previous reports implicating that mutant p53 is conferring radio-resistance in various cancer types when tested with low-LET type of irradiation [58, 59]. The presence of mutant p53 has been associated with impaired DNA damage response, compromised cell cycle checkpoints collectively contributing to the decreased sensitivity to RT [29, 60, 61]. Our findings suggest that in high-LET alpha particle-based RT, tumors harboring mutant p53 are profoundly affected and that reactivation of the WT form in such tumors could be instrumental in augmenting the effect of the RT even further. These findings emphasize the clinical relevance of such modalities in patients carrying hotspot mutations in p53 and further support the notion that the ability of APR-246 to reactivate p53 might differ between the GOF mutants.

To address the challenge posed by mutant p53, we explored the potential of APR-246 as a pharmacological agent restoring p53 function. Our data revealed that APR-246 treatment effectively restored p53 function in cancer cells with mutant p53, as evidenced by the reestablishment of p53 target gene expression and subsequent downstream effects on cell cycle regulation and apoptosis induction. The combined treatment of APR-246 and ^224^Ra sources resulted in a substantial reduction in tumor volumes and increased rates of tumor eradication compared to either treatment alone. When HCT116 tumors lacking p53 or carrying the WT p53 form were treated with the combination of ^224^Ra source and APR-246 (Supplementary Fig. 5), we did not observe any significant difference in tumor growth rate compared to the RT alone. These findings strongly indicate that APR-246, given the preceding conditions of DNA damage caused by the alpha-particles-based RT, augments the response via p53 reactivation.

The mechanisms underlying the combined effects of APR-246 and alpha-emitting atoms can be attributed to multiple factors. First, restoration of p53 function by APR-246 may reinstate the ability cells in the periphery of the radioactive source’ ‘kill zone’ to recognize and repair DNA damage induced by high-LET radiation, thereby promoting the activation of efficient DNA repair pathways. Second, APR-246-mediated reactivation of p53 signaling may enhance cell cycle arrest and apoptosis in response to radiation-induced DNA damage, leading to increased tumor cell death. Additionally, the reestablishment of p53-mediated transcriptional regulation by APR-246 may restore the expression of key genes involved in cellular response to radiation, such as those associated with DNA repair, cell cycle control, and apoptosis.

While APR-246 was initially characterized as a reactivator of mutant p53 function, its p53-independent mechanisms of action have garnered increasing attention and growing evidence suggests that APR-246 exerts its anti-cancer effects through p53-independent mechanisms as well [62–64] (). These include the induction of oxidative stress and the depletion of cellular glutathione levels, leading to the activation of apoptosis in cancer cells irrespective of their p53 status. Furthermore, recent preclinical studies have identified additional molecular targets of APR-246, such as heat shock protein 70 (HSP70) and the proteasome, which are involved in protein folding, degradation, and cellular stress responses. In addition and on the same note, accumulating research has unveiled APR-246 interactions with the p73 and p63 proteins, two structural homologs of p53, which are known to play critical roles in cellular homeostasis and tumorigenesis [38] (). Since p73 and p63 proteins share significant structural and functional similarities with p53, APR-246 interacts with both proteins. APR-246 was shown to promote the expression of target genes involved in cell cycle regulation, apoptosis, and DNA repair [65–67]. It is reasonable to hypothesize that APR-246 may indirectly affect the function of both TAp73 and TAp63 isoforms. Previous reports indicate that mutant p53 mutants can inhibit TAp73 and TAp63 by a direct oligomerization via the core domain. Potentially, APR-246 may interfere with the protein–protein interaction between p53 mutants and TAp73/TAp63, thereby preventing their inhibitory effect. Additionally, since TAp73 can enhance p53 transcriptional activity, it is possible that co-expression of high levels of TAp73 may enhance the effect of APR-246 on mutant p53 reactivation. Further investigation is required to assess the involvement of p53 family members when cells are pre-exposed to alpha particles and then treated with APR-246.

It is worth noting that while our study provides compelling evidence for the efficacy of APR-246 in combination with alpha-particles-based RT, further preclinical and clinical investigations are warranted to establish the safety, optimal dosage, and treatment schedule of APR-246 in combination with alpha-particle-based RT. Moreover, exploring the potential of this combination therapy in various cancer types and identifying specific biomarkers that predict responsiveness to APR-246 and RT will be crucial for future translational and personalized medicine applications.

As mentioned above, since there are also p53-independent mechanisms of response to DNA damage, additional apoptosis-inducing compounds could be considered to be conjugated with alpha-particle-based treatments. Apoptosis-inducing agents have emerged as promising treatments for cancer, aiming to trigger programmed cell death in cancer cells [68, 69]. Several clinical trials are currently investigating the efficacy and safety of these agents, including compounds such as BH3 mimetics, BCL-2 protein inhibition, Smac mimetics, and CD95 agonists, in diverse cancer types, with encouraging preclinical outcomes demonstrating their potential as novel therapeutic interventions [70–72].

In conclusion, our study demonstrates that cancer cells with mutant p53 exhibit radiosensitivity to alpha-particles-based radiotherapy. We provide evidence that APR-246 effectively enhances the sensitivity of mutant p53 cancer cells and tumors to alpha-particles-based RT. These findings offer a promising therapeutic approach for improving treatment outcomes in patients with mutant p53 tumors. Future studies should focus on further elucidating the mechanisms underlying the synergistic effects of APR-246 and radiotherapy and translating these findings into clinical settings.

## Materials and methods

### Cell culture

All cell lines were grown and maintained as per ATCC guidelines. In general, PANC-1 pancreatic carcinoma cells harboring the R273H p53 mutant where the mutant p53 was knocked out (a gift from the group of Prof. Moshe Oren, Weizmann Institute of Science, Israel) as described in [54] were grown in Dulbecco’s Modified Eagle Medium (DMEM) (Gibco (Thermo-Fisher), Waltham, MA, USA) and HCT116 colon carcinoma cells were used as an isogenic set of 3 lines differing by their p53 status: p53(+/+), p53 (−/−) and p53 (-/R248W) mutant. (From the group of Bert Vogelstein, Johns Hopkins Hospital, USA) were maintained in McCoy medium (Gibco (Thermo-Fisher), Waltham, MA, USA). HT-29 cells and the shp53 derivate were also provided by the group of Prof. Moshe Oren at the Weizmann Institute of Science, Israel. To create the stable knock-down of mutant p53, the cells were infected with recombinant lentiviruses (pLKO.1-puro-shp53, (addgene, 19199) to produce shRNA directed against the endogenous mutant p53 mRNA. p53 knockdown and mutant protein expression were verified by RT-qPCR and Western blot analysis as also presented in [73]. The amount of 10% fetal bovine serum (FBS) (Gibco (Thermo-Fisher), Waltham, MA, USA) and 1% Penicillin (100 U/mL) and streptomycin (100 U/mL) (Gibco (Thermo-Fisher), Waltham, MA, USA) were included in all media. Cells were grown at 37 °C supplemented with 5% CO2. All cell lines were diluted twice a week and used until passage 15. All lines were tested for mycoplasma contamination and authenticated using an STR profiling (unless bought directly from the ATCC recently).

### α irradiation

This set-up was described also in [45]. Briefly, cells seeded on a thin (7.5 µm) kapton (polyimide) foil were irradiated by alpha particles traversing the foil from below. The kapton foil (Dupont, Luxembourg) was placed between the 2 rings. Cells were seeded on the foil at a density of 3·10^4^ cells per well and were exposed to the alpha particle flux 24 h later. Exposure was performed by positioning the cells seeded on the foil 9.8 mm above a silicon wafer coated with a thin layer of ^241^Am in secular equilibrium with its daughters in air. HCT116 cells were seeded in kapton wells, as described above, and treated either with or without the APR-246 treatment. Four hours after any treatment, cells were collected using trypsin and use for further in vitro assays.

### Colony formation assay

Following irradiation, cells were harvested and sparsely seeded in 6-well plate (Corning, Corning, NY, USA) with 2 ml of medium in each well, to allow the formation of well-separated colonies. Colony formation was monitored daily, and typically 14 days post irradiation colonies were fixed in methanol and stained with crystal violet staining. To replace the time-consuming manual colony counting protocol, a semi-automatic approach was addressed; we utilized a technologically advanced scanning microscope, CytoSMART Omni FL (by Axion Biosystems, Inc.), capable of providing high-resolution images of colonies in wells with high throughput. Plate images can be analyzed offline by appropriate colony counting software packages, and their high resolution provides the capability of simply zooming into a colony to verify whether it consists of more or less than 50 cells. High-resolution images were further analyzed by ImageJ software [74] with basic image processing functions like contrast and color thresholds to reduce the background noise parallel to enhancing the colony pixels, as well as the *watershed* function as an attempt to separate overlapping colonies. In addition, we apply the *Analyze particles* function to generate colony contours, assigning each contour an index (i.e., a count number) and summarizing them in a list. At this stage, the analyst only needs to make decisions for each colony in the list, such that the decision can be verified by observing the original image and magnifying to the regions of interest.

### RNA and real-time quantitative PCR

RNA was isolated with a mini-RNeasy kit (Qiagen, Germany) and reverse transcribed using Moloney murine leukemia virus reverse transcriptase and random hexamer primers (Promega, USA). Real-time qPCR was performed using SYBR Green Master Mix (Thermo Fisher) in a StepOnePlus instrument (Applied Biosystems). All primers were purchased from Sigma-Aldrich. Each sample was analyzed in triplicate and data were analyzed based on the comparative Ct (2 − ΔΔCt) method. The expression of target genes was normalized to GAPDH expression. The primers used in this study are detailed here:

NOXA: F: 5′-GAAGGGAGATGACCTGTGATTAG-3′/R:5′-TGCTGAGTTGGCACTGAAA-3′.

PUMA: F: 5′-GGA GCA GCA CCT GGA GTC / R: 5′-TA CTG TGC GTT GAG GTC GTC-3′.

GAPDH: F:5′-GGTGTGAACCATGAGAAGTATGA-3′/R:5′-GAGTCCTTCCACGATACCAAAG-3′.

### Tumor inoculation

The study was approved by the Ben-Gurion University Institutional Animal Care and Use Committee and was conducted according to the Israeli Animal Welfare Act following the guidelines of the Guide for Care and Use of Laboratory Animals (National Research Council, 1996) [permit no. IL-47-07- 2019(E)]. Male nude mice (6–12 weeks old) were obtained from Envigo, Israel. Mice were inoculated subcutaneously with 5·10^6^ cells (for all cell lines) in 100 μl Dulbecco’s phosphate-buffered saline (DPBS and DMEM) (Gibco, 14190144, Thermo Fisher Scientific, MA, USA) into the low lateral side of the back. At the day of source insertion and before the insertion, the mice were divided into treatment groups to create a similar as possible mean tumor volume for each group. Blinding was not conducted.

### Ethics approval and consent to participate

No human subjects were used in this study, therefore ethics and consent are not applicable.

### Frozen section preparation

Four to five days post the ^224^Ra treatment, the tumors were excised (as a whole). Each tumor was cut to two halves, in the estimated location of the seed center, perpendicular to the seed’s insertion axis. The seed was then pulled out using surgical tweezers and was placed in a 1.5 ml microcentrifuge tube filled with 1 ml of water. The tumor was placed for 1 h in −80 °C. The tumors were put in 20 ml santilation bottles on dry-ice and taken for a measurement with HIDEX gamma. Subsequently, both halves of the tumor were subjected to histological sectioning by LEICA CM 1520 cryostat (Buffalo Grove, IL, USA). The 10 µm-thick sections were then placed on positively charged glass slides (76 mm by 26 mm by 1–1.2 mm), with 250–300 µm intervals between each section, creating a series of sequential sections (between 5 and 15 per tumor). Following the sectioning, slides were fixed with 4% paraformaldehyde (sc-281692, Santa Cruz Biotechnology Inc., Dallas, TX, USA) for 10 min and rinsed twice with PBS for 10 min each time. Immediately after the fixation step, slides were taken to the autoradiography system. The same histological sections measured on the imaging plate, were later stained with hematoxylin-eosin (Surgipath, Richmond, IL). The pictures were taken using a Panoramic scanner (3D HISTECH Ltd, Budapest, Hungary).

### ^224^Radium‑loaded source preparation and insertion

Stainless steel seeds (0.1 mm in diameter, cut to a length of 6.5 mm) were loaded with ^224^Ra atoms (half-life of 3.7 days). To prevent Radium dissolution in the tissue fluids, the atoms were embedded a few atomic layers into the seed surface through thermal treatment. Seeds, either loaded with ^224^Ra or inert, were placed near the tip of an 18-gauge needle attached to a 2.5 ml syringe (Picindolor, Rome, Italy) and inserted into the tumor by a plunger placed internally along the syringe axis. The radioactive and inert seeds were inserted into the primary tumor under anesthesia with Isoflurane. Seed location was verified using a Geiger counter (RAM GENE-1, Rotem industries, Israel) after insertion process was completed and before tumor removal.

### Tumor volume measurements

Local tumor growth was determined by measuring three mutually orthogonal tumor dimensions three times per week, according to the following formula: Tumor volume = length·weight·height·*π*/6. Mice were pre-excluded from the study based on tumor non-uniformity criteria (too big/small tumors before source insertion, double focal tumors, internal tumors) and if the source fell in first 5 days. Tumor volume over time was assessed and compared between the groups using repeated measures ANOVA analysis. The cubic root transformed volume was modeled as a function of group, day (categorical) and the day × group interaction with baseline volume entered as a covariate. Mean (least squares means) and confidence intervals were estimated from the interaction term for each day per group and were back transformed to the volume.

Survival data was depicted by a Kaplan–Meier plot; two curves were compared with a Log-rank test with p-values adjusted for multiple comparisons using the FDR method.

### Drug preparation, storage and administration

APR-246 was purchased from Cayman Chemicals was dissolved in DPBS on the same day of the experiment. The stock was stored at −20 °C in powder condition. For the in vivo studies, APR-246 was dissolved DPBS, and 100 µl of 50 mg/kg APR-246 was injected *i.p*. twice a day; DPBS was used as a sham control. Treatment started 1 day after ^224^Ra source insertion (day 0) for a total of fourteen doses (days 1–7).

### Spatial apoptosis assay

The DeadEnd™ Fluorometric TUNEL System (Promega) measures the fragmented DNA of apoptotic cells by catalytically incorporating fluorescein-12-dUTP at 3′-OH DNA ends using Terminal Deoxynucleotidyl Transferase (TdT), which forms a polymeric tail using the principle of the TUNEL (TdT-mediated dUTP Nick-End Labeling) assay. The fluorescein-12-dUTP-labeled DNA can then be visualized directly by fluorescence microscopy or quantitated by flow cytometry. Imaging was done on Zeiss Cell Discovery 7 system, equipped with a Plan-Apochromat 20×/0.95 objective lens and appropriate LED and filter configuration. Subsequent image analysis was conducted in QuPath software version 0.4.4. Initially, manual annotations, delineating the cancerous regions and the position of the capsules, were created for each slide. The cancer regions (ROIs) were subdivided into 100 μm^2^ tiles utilizing the built-in SLIC superpixel segmentation algorithm. The mean number of superpixels within each ROI was estimated at 500 ± 75, with a mean analyzed area of 0.5 ± 0.1 mm^2^. Quantitative descriptors capturing the intensity of the green channel were calculated for every superpixel, followed by the visualization of the average intensities of these superpixels as a heat map superimposed onto the images. Statistical analysis was performed using GraphPad Prism 10. Preliminary assessment of normal distribution was carried out using the Shapiro-Wilk test. The Wilcoxon nonparametric test was employed to evaluate the statistical variance between the control and AFP groups.

### Autoradiography of ^224^Ra-treated tumors and ^212^Pb leakage probability measurements

A single ^224^Ra seed (6.5 mm length, 0.7 mm outer diameter), carrying 3 μCi ^224^Ra, was inserted to the center of a mice-borne HCT116 or PANC-1 tumors. Four to five days later, the tumor was excised (as a whole) and cut in two halves, at the estimated location of the seed center, perpendicular to the seed axis. The seed was then pulled out using surgical tweezers and placed in a water-filled tube for subsequent measurement by a well-type NaI(Tl) detector (Hidex Automatic Gamma Counter). The tumor was kept for 1 h at −80 °C. It was then taken, in dry ice, for measurement in the same gamma counter to determine the ^212^Pb activity it contains, by focusing on the ^212^Pb 239 keV gamma line. The measurements of the seed and tumor activity were used to determine the ^212^Pb leakage probability from the tumor (i.e., the probability that a ^212^Pb atom released from the seeds leaks out from the tumor through the blood before its decay).

Immediately after the gamma measurement, both halves of the tumor were subjected to histological sectioning using a LEICA CM 1520 cryostat (Buffalo Grove, IL, USA). Sections were cut at 250–300 μm intervals with a thickness of 10 μm, and were then placed on positively charged glass slides, fixed with 4% paraformaldehyde (sc-281692, Santa Cruz Biotechnology Inc., Dallas, TX, USA) and rinsed twice with PBS. Typically, there were 5–15 sections per tumor, spanning a length of 1.5–5 mm. Shortly after their preparation, the glass slides were placed, faced down, for a duration of 1 h, on a phosphor imaging plate (Fujifilm TR2040S) protected by a 12-μm Mylar foil and enclosed in a light-tight casing. Alpha particles emitted from the sections in the decays of ^212^Pb progeny atoms, ^212^Bi and ^212^Po, penetrate through the foil and deposit energy in the active layer of the phosphor imaging plate. Immediately after exposure, the plate was read out by a phosphor-imaging scanner (Fujifilm FLA-9000). The intensity (in units of photo-stimulated luminescence) was converted to ^212^Pb activity using suitable ^212^Pb calibration samples. By calculating the total area corresponding, in a given tumor section, to an asymptotic ^212^Bi/^212^Po alpha dose larger than 10 Gy, the effective diameter is defined by: deff = 2[A(DBiPo > 10 Gy)/π]1/2. The 10-Gy dose is chosen as a convenient reference for actual therapeutic alpha-particle doses that are expected to be in the range ~10–20 Gy. The same histological sections measured on the imaging plate were later stained with hematoxylin–eosin (H&E) (G-biosciences, St Louis MO, USA) for tissue damage detection. H&E staining was correlated with the activity distribution measurements. The pictures were taken using a Panoramic scanner (3D HISTECH Ltd., Budapest, Hungary).

### Western blotting

Cells were lysed with 30 µL of RIPA 1× buffer supplemented with Protease inhibitor cocktail (1:100, Thermo Fisher Scientific, Waltham, MA, USA). Protein concentration in the lysates were determined via the Pierce BCA protein assay kit (Thermo Fisher Scientific, Waltham, MA, USA). Lysates were mixed with sample buffer (5×) (Thermo Fisher Scientific, Waltham, MA, USA) and boiled at 95 °C for 10 min, loaded into gel (Invitrogen Novex WedgeWell 4 to 20%, Tris-Glycine, 1.0 mm, Mini Protein Gel, 10-well, Thermo Fisher Scientific, Waltham, MA, USA), and separated via PAGE. Proteins were then transferred into the nitrocellulose membrane (Greiner, 10-6000-02), blocked for 1 h at room temperature with 5% BSA in TBS and then followed by exposure to primary antibodies (dil 1:1000) overnight at 4 °C: anti-p53, anti-p21, and anti-GAPDH. The membranes were then washed and incubated with HRP-conjugated secondary antibody (dil 1:5000) for 1 h at room temperature, washed, and exposed to SuperSignal West Pico PLUS Chemiluminescent Substrate (Thermo Fisher Scientific, Waltham, MA, USA) as per manufacturer’s protocol before visualizing the membranes in iBrightCL1000 (Invitrogen, A32749, Carlsbad, CA, USA).

### Antibodies

Mouse Monoclonal anti-p53 (DO-1) was purchased from Santa Cruz Biotechnology, Santa Cruz, CA, USA. Mouse anti-GAPDH was purchased from Sigma-Aldrich, St. Louis, MO, USA. Anti-p21 antibody [EPR362] was purchased from abcam, Cambridge Biomedical Campus, Hills Road, Cambridge. Anti-Cleaved Caspase-3 antibody (Ab-2302) was purchased from abcam, Cambridge Biomedical Campus, Hills Road, Cambridge.

### Biostatistical analysis

All statistical tests were performed in GraphPad PRISM 8.0. and presented as the mean ± standard of measurement. Continuous variables were compared by using the Student’s t-test. Categorical variables were compared by using Chi-square (or the Fisher exact test when appropriate). No statistical methods were used to predetermine the sample size. The variance was similar between the groups that were being statistically compared. Tumor volume over time was assessed and compared between the groups using repeated measures Two-way ANOVA unless stated otherwise. Each experiment was analyzed until the time point at which the first animal died. Survival curves are depicted by a Kaplan–Meier plot and compared with a Log-rank test. A *p*-value < 0.05 was considered statistically significant.

### Supplementary information

Supplemental Material

Blots

## Data Availability

All materials described in the manuscript, including all relevant raw data, will be freely available to any researcher wishing to use them for non-commercial purposes, without breaching participant confidentiality.
