# Supplementary material for: APR-246 as a radiosensitization strategy for mutant p53 cancers treated with alpha-particles-based radiotherapy
Source: Cell Death Dis. 2024 Jun 18;15(6):426. doi: 10.1038/s41419-024-06830-3 (PMC11189442; doi:10.1038/s41419-024-06830-3)
Supplement: Supplementary file 1 — Supplemental Material [file 41419_2024_6830_MOESM1_ESM.docx]

**Supplementary materials:**

**APR-246 as a radiosensitization strategy for mutant p53 cancers treated with alpha-particles-based radiotherapy**

We display here 6 figures not included in the main text:

1.The decay chain of ^224^Ra

2. A representative photo of mice affiliated with main Figure 2.

3. Alpha particles and APR-246 combination increase cell death in mutant p53 cancer cells, affiliated with main Figure 3.

4. Tumor growth of groups of mice bearing HCT116 or PANC-1 large tumors, affiliated with main Figure 4

5. Tumor growth of groups of mice bearing HCT116 tumors with WT or NULL p53, affiliated with main Figure 4

6. Graphical representation of the suggested model


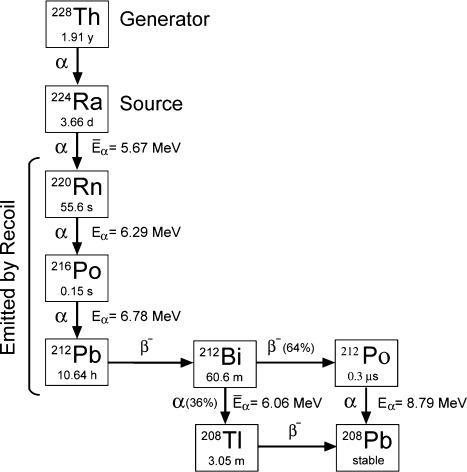


**Supp. Figure 1.** The decay chain of ^224^Ra.


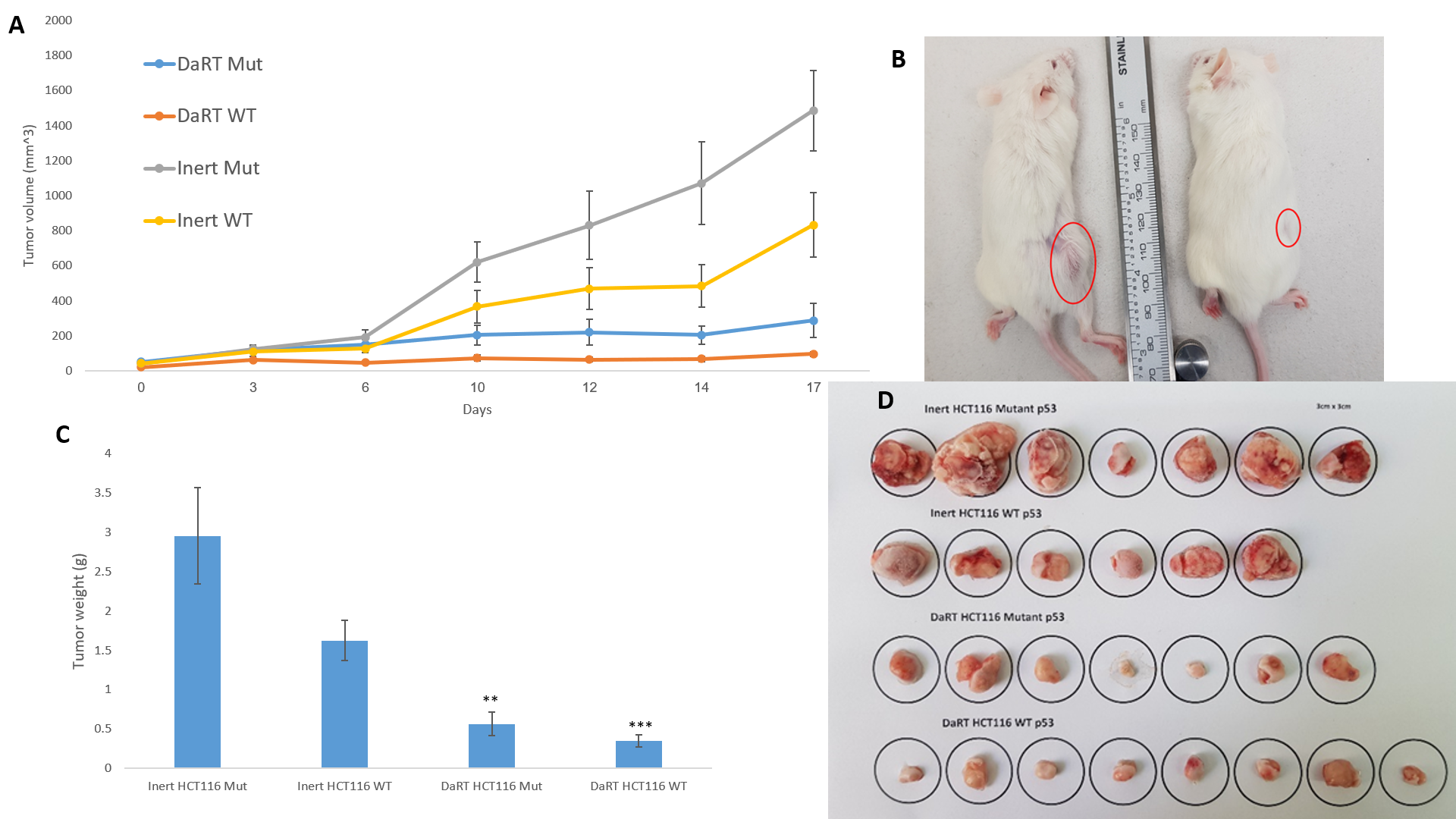


**Supp. Figure 2. Tumors carrying GOF p53 mutants are more resistant to high-LET RT.**

HCT116 tumors differing by p53 status (WT and Mut) were transplanted as xenografts, treated with either Inert or ^224^Ra sources and monitored for tumor growth and weight. The figure above shows representative photos of Inert (left) or ^224^Ra-source (right) treated animals 17 days post-treatment.

IR

**A**

**PANC-1**

**HT-29**


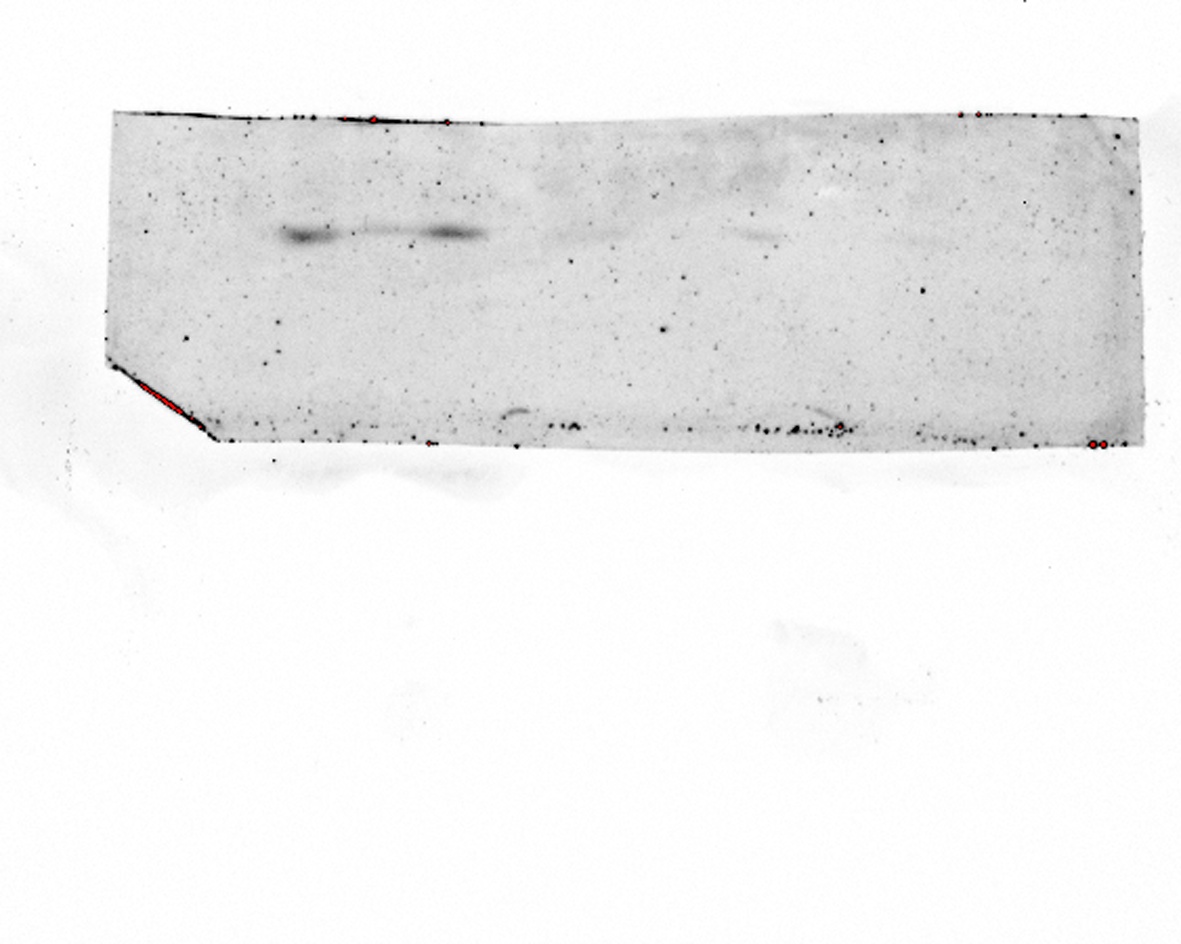

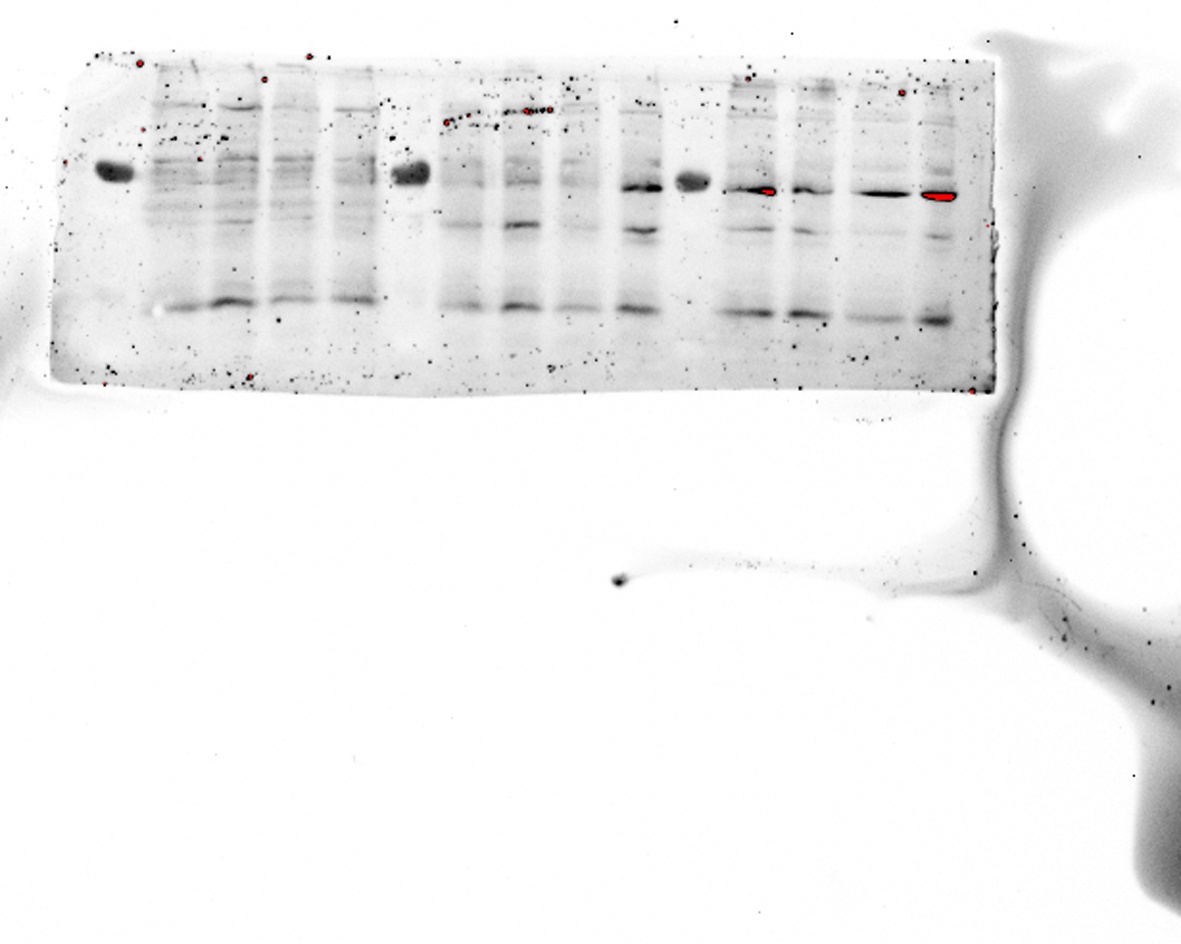

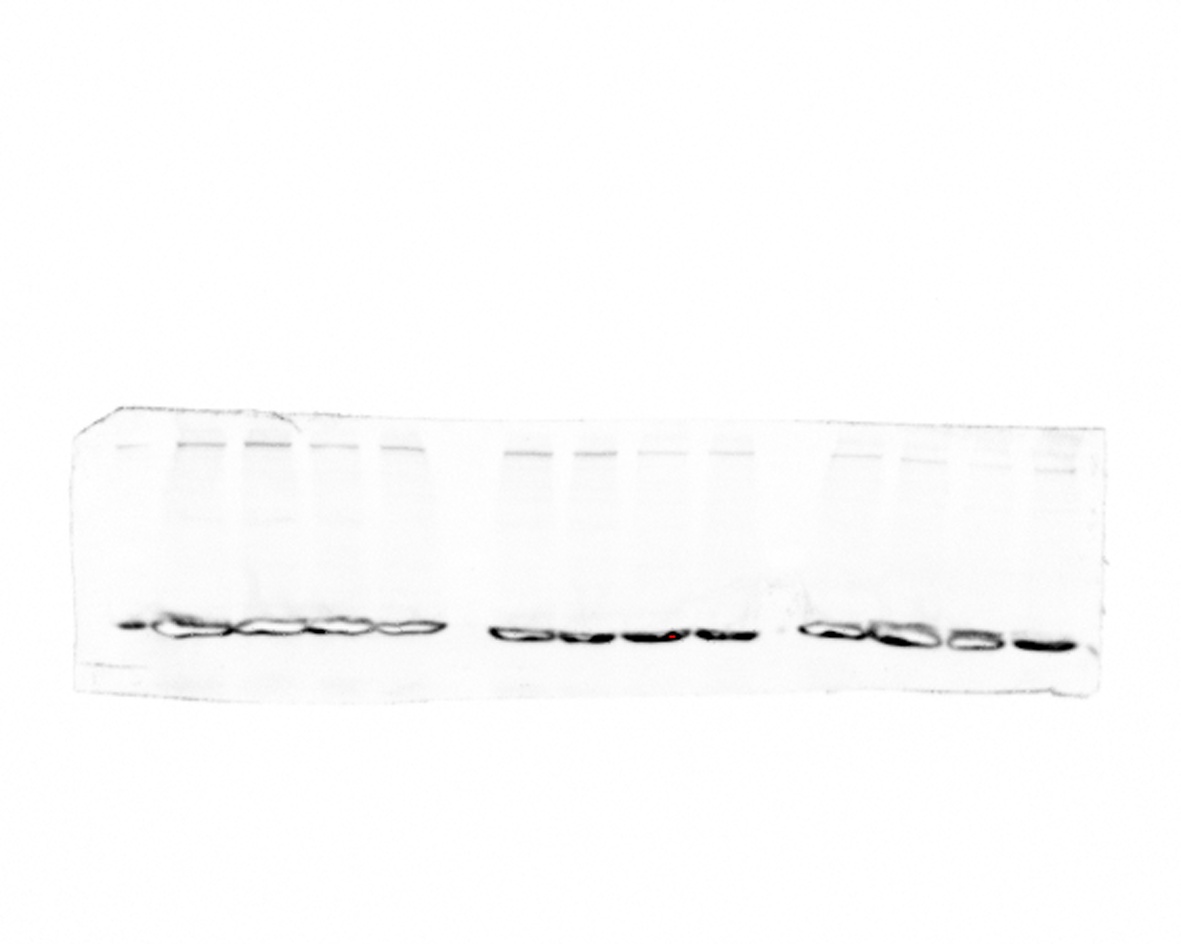

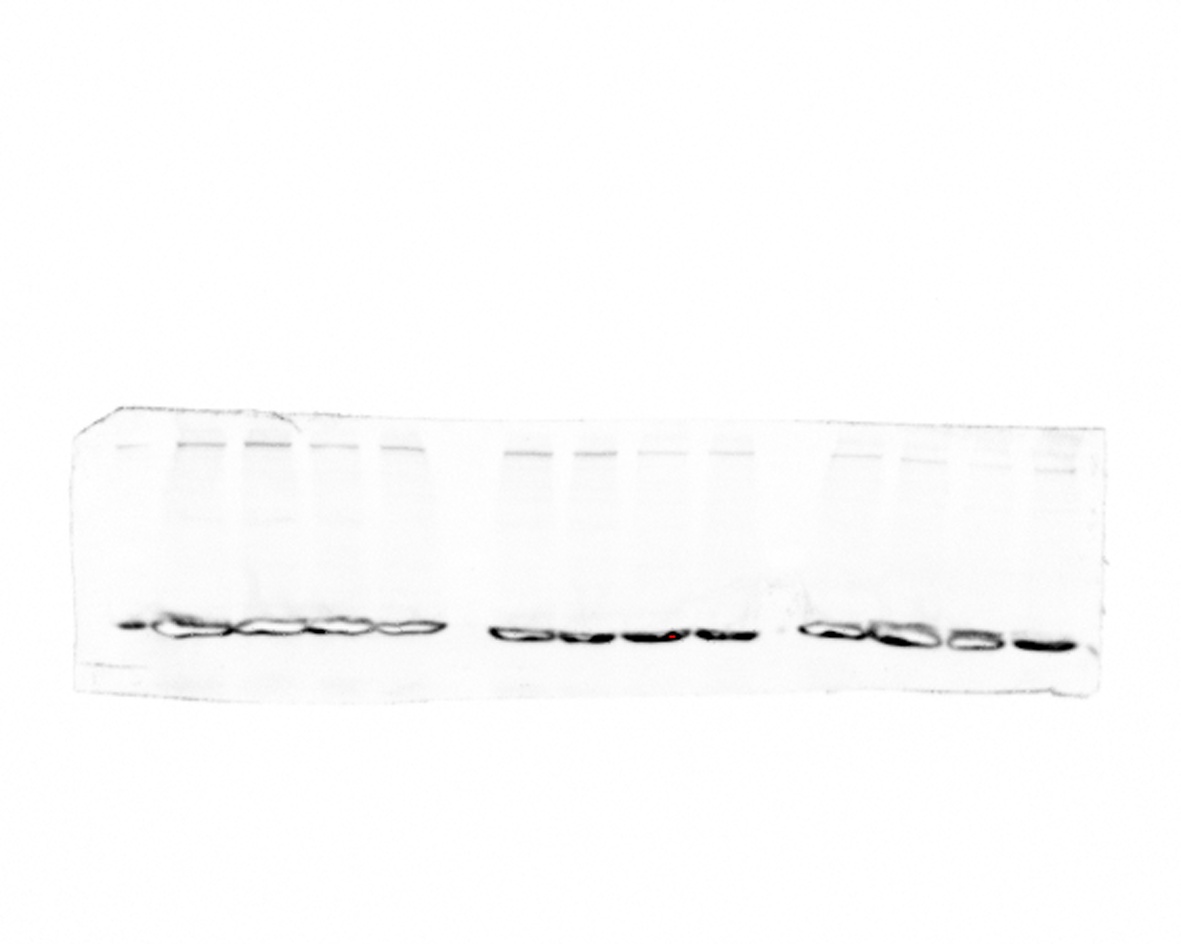

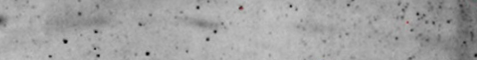

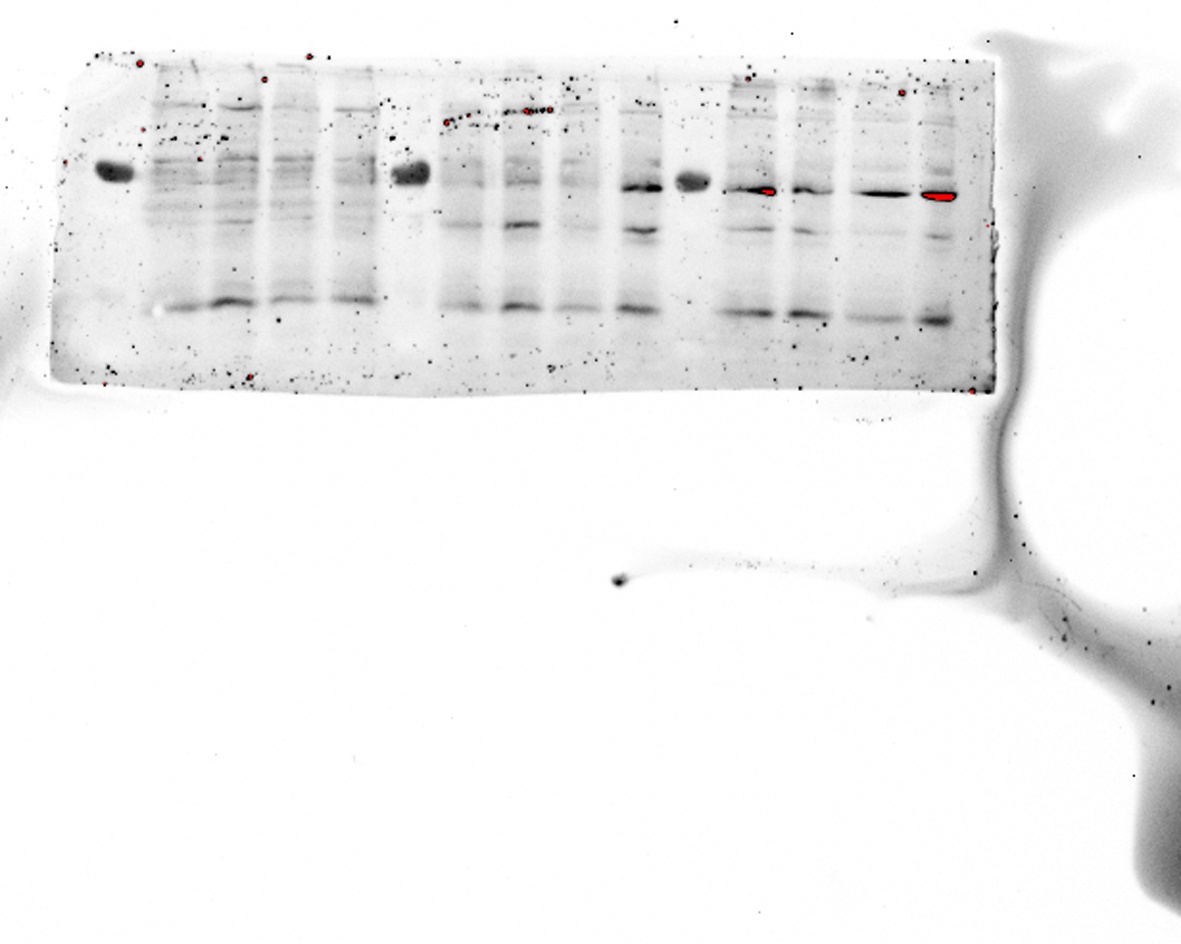


**- - + +**

**- - + +**

**- + - +**

**Beta-Actin**

**CASPASE-3**

**NOXA**

**Beta-Actin**

**CASPASE-3**

**- + - +**

**NOXA**

**APR**

**IR**


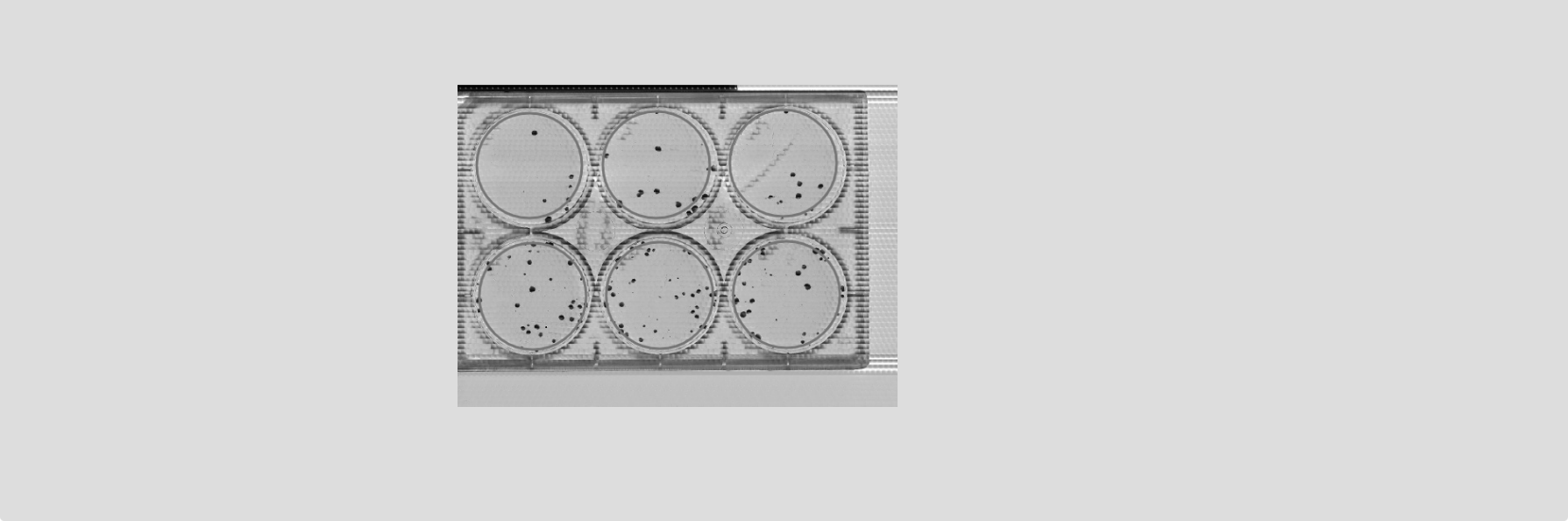

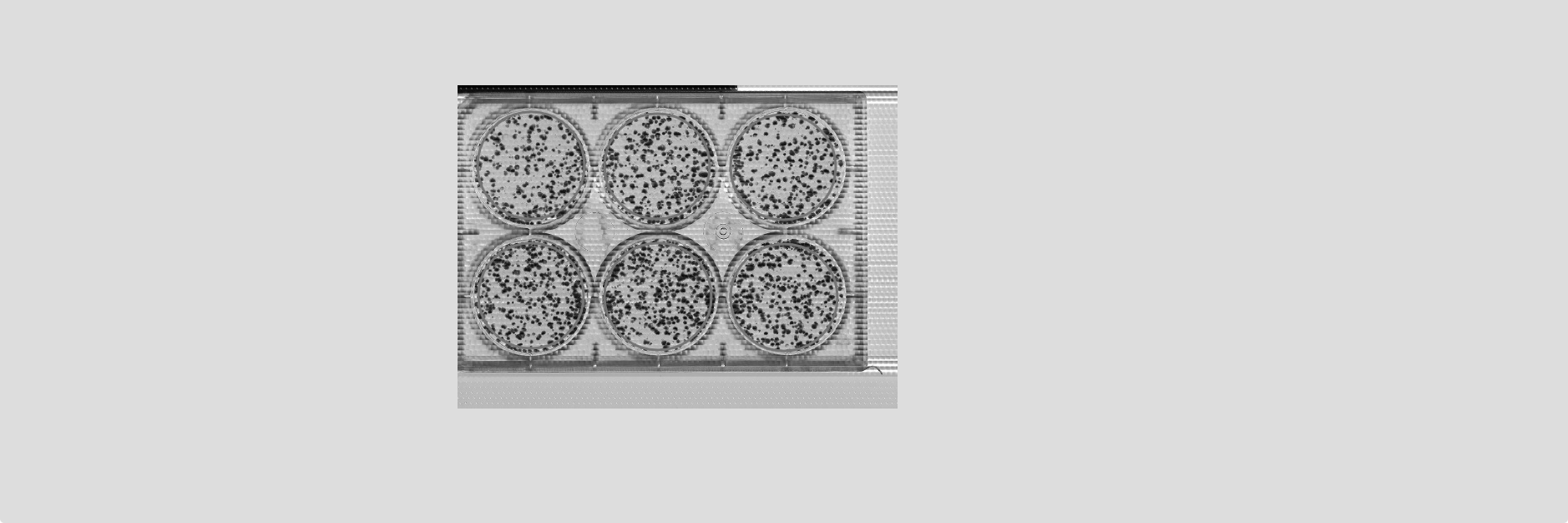

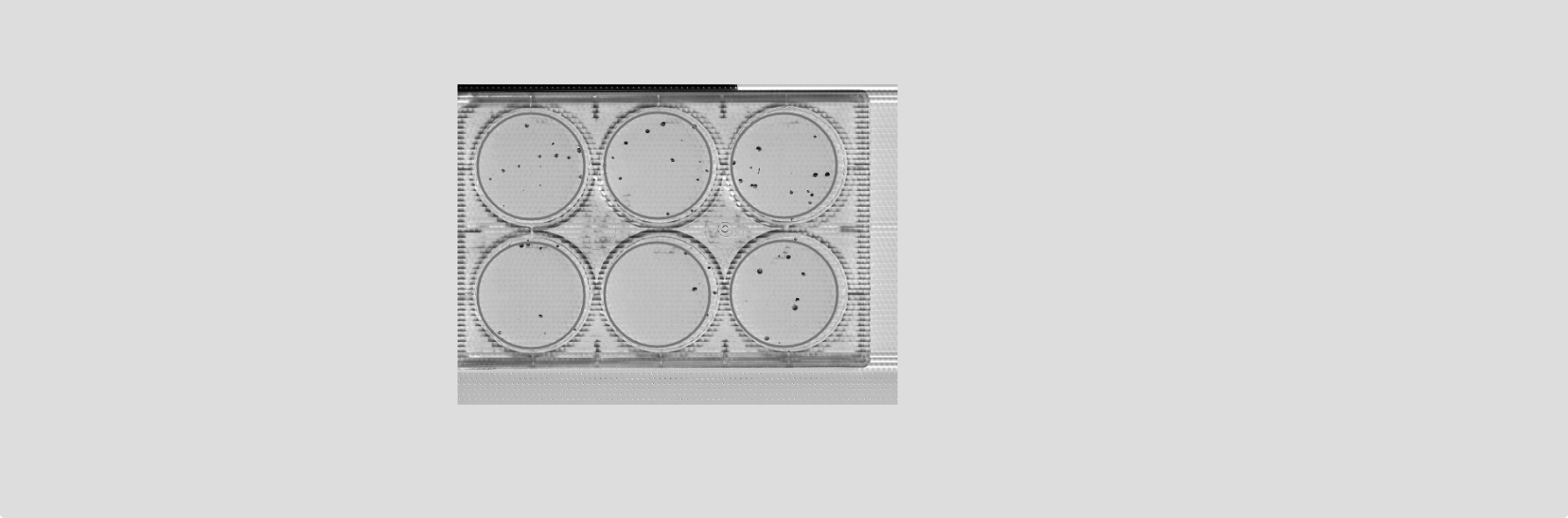

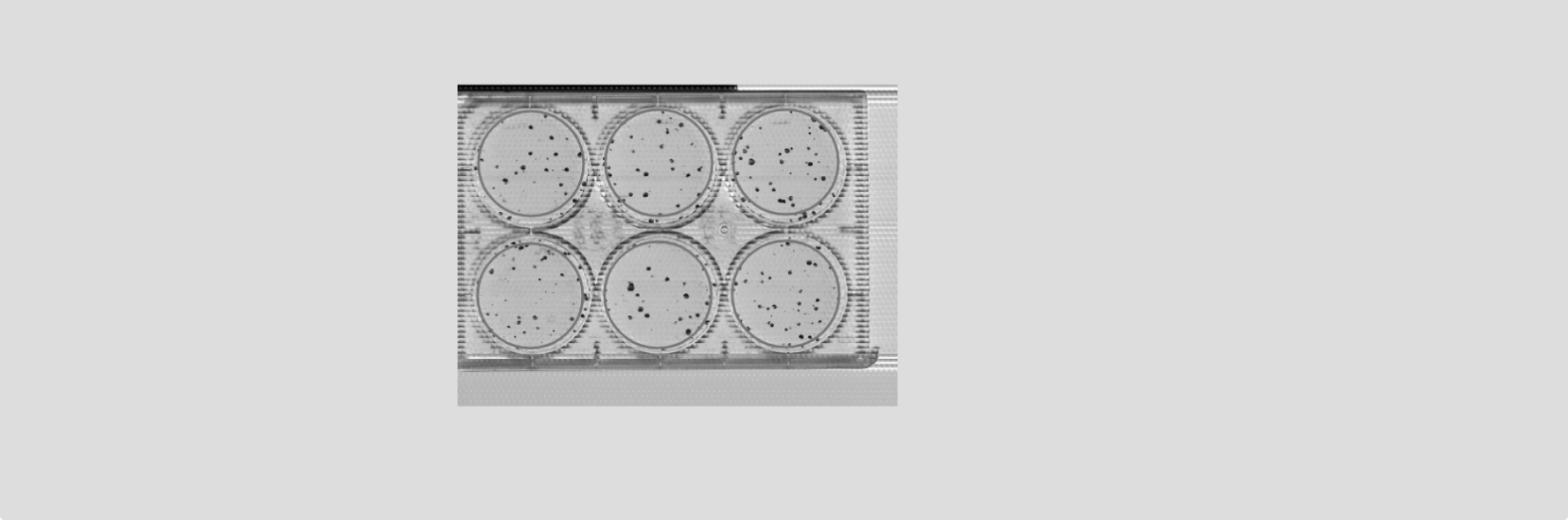


**B**

**APR**

**Control**

**IR + APR**

**IR**

**C**


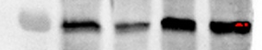

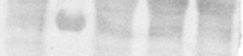

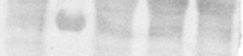

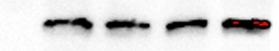

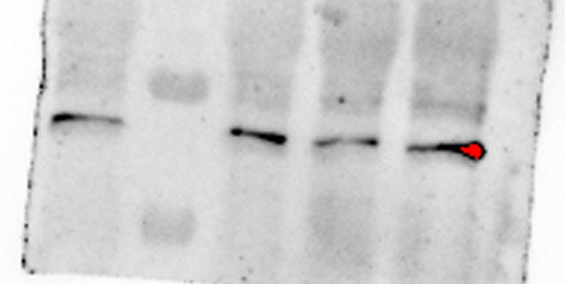

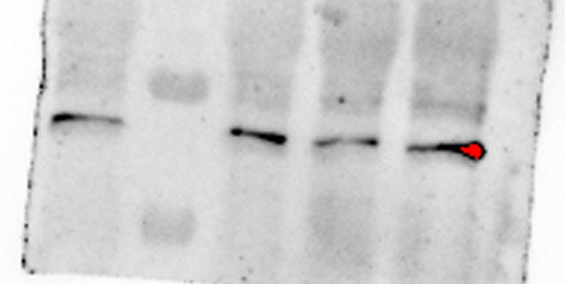

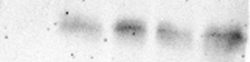

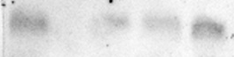

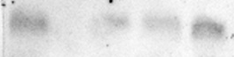


**D**

**Beta-Actin**

**HCT116 p53 MUT**

**HCT116 p53 -/-**

**APR-246**

**IR**

**- + - +**

**- - + +**

**p53**

**p21**

**- + - +**

**- - + +**

**Supp. Figure 3.** **Alpha radiation combined with p53 reactivation yielded an increase in apoptosis and radio-sensitivity.**  (A) PANC-1 (left panel) and HT-29 cells (right panel) were treated with APR-246 (25µM, 8hrs), or irradiated with 1Gy of X-rays (IR), or both. Proteins were extracted and levels of CASPASE-3 and NOXA were evaluated, as Beta-Actin served as a loading control. (B) Colony formation assay of HCT116 mutp53 cells treated with 1Gy of alpha irradiation (IR), 25µM of APR-246 (APR-246) or a combination (IR+APR-246), compared to a non-treated control. (C) Cell viability assay for PANC-1 cells either irradiated with 1Gy of alpha irradiation (IR), treated with APR-246 (APR-246) or a combination (IR+Apr-246). (D) HCT116 cells, either MUT (left panel) or p53 -/- (right panel) were treated with APR-246 (25µM, 8hrs), or irradiated with 1Gy of alpha particles (IR), or both. Proteins were extracted and levels of p53 and p21 were evaluated, as Beta-Actin served as a loading control.

**A**

**B**

**Supp. Figure 4.** **Combination of high-LET ^224^Ra source with APR-246 in large CRC and PDAC xenografts** (A) Mean tumor volume ± SEM of HCT116-bearing mice (~200 mm^3^ average volume) were treated with a 75-kBq ^224^Ra source or inert source on day 0, followed by 14 doses of 50 mg/kg APR-246 i.p. on days 1–7. (*** p < 0.005). (B) Mean tumor volume ± SEM of HCT116-bearing mice (~100 mm^3^ average volume) were treated with a 75-kBq ^224^Ra source or inert source on day 0, followed by 14 doses of 50 mg/kg APR-246 i.p. on days 1–7.

**A**


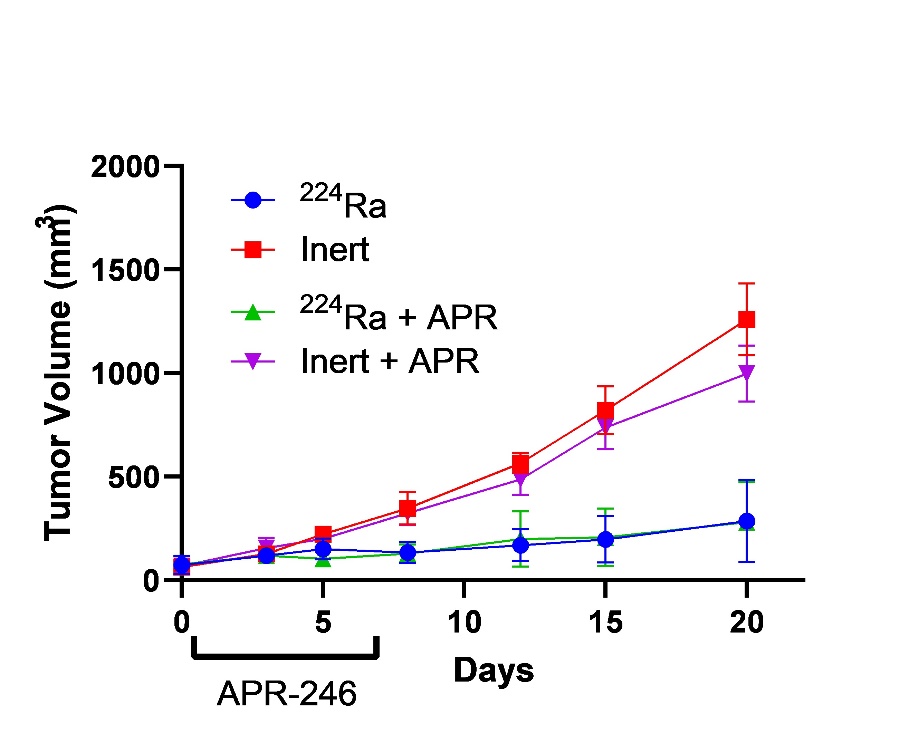


**HCT116 p53 (+/+)**

**B**

**HCT116 p53 (-/-)**


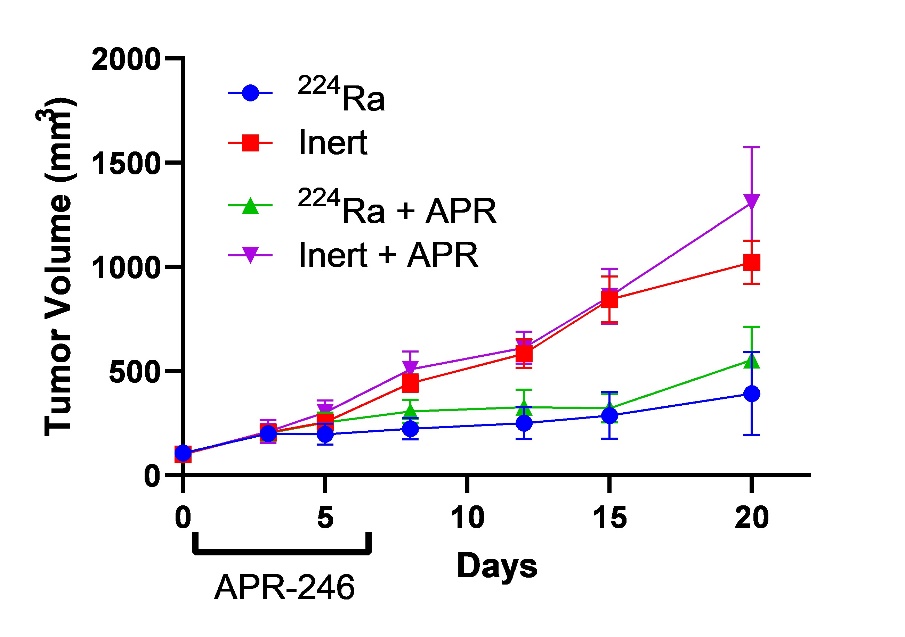


**Supp. Figure 5.** **Combination of ^224^Ra source with APR-246 in HCT116 xenografts carrying either WT or NULL p53** (A) Mean tumor volume ± SEM of WT p53 (+/+) HCT116 -bearing mice (~75 mm^3^ average volume) were treated with a 75-kBq ^224^Ra source or inert source on day 0, followed by 14 doses of 50 mg/kg APR-246 i.p. on days 1–7. (B) Mean tumor volume ± SEM of NULL p53 (-/-) HCT116 -bearing mice (~75 mm^3^ average volume) were treated with a 75-kBq ^224^Ra source or inert source on day 0, followed by 14 doses of 50 mg/kg APR-246 i.p. on days 1–7.


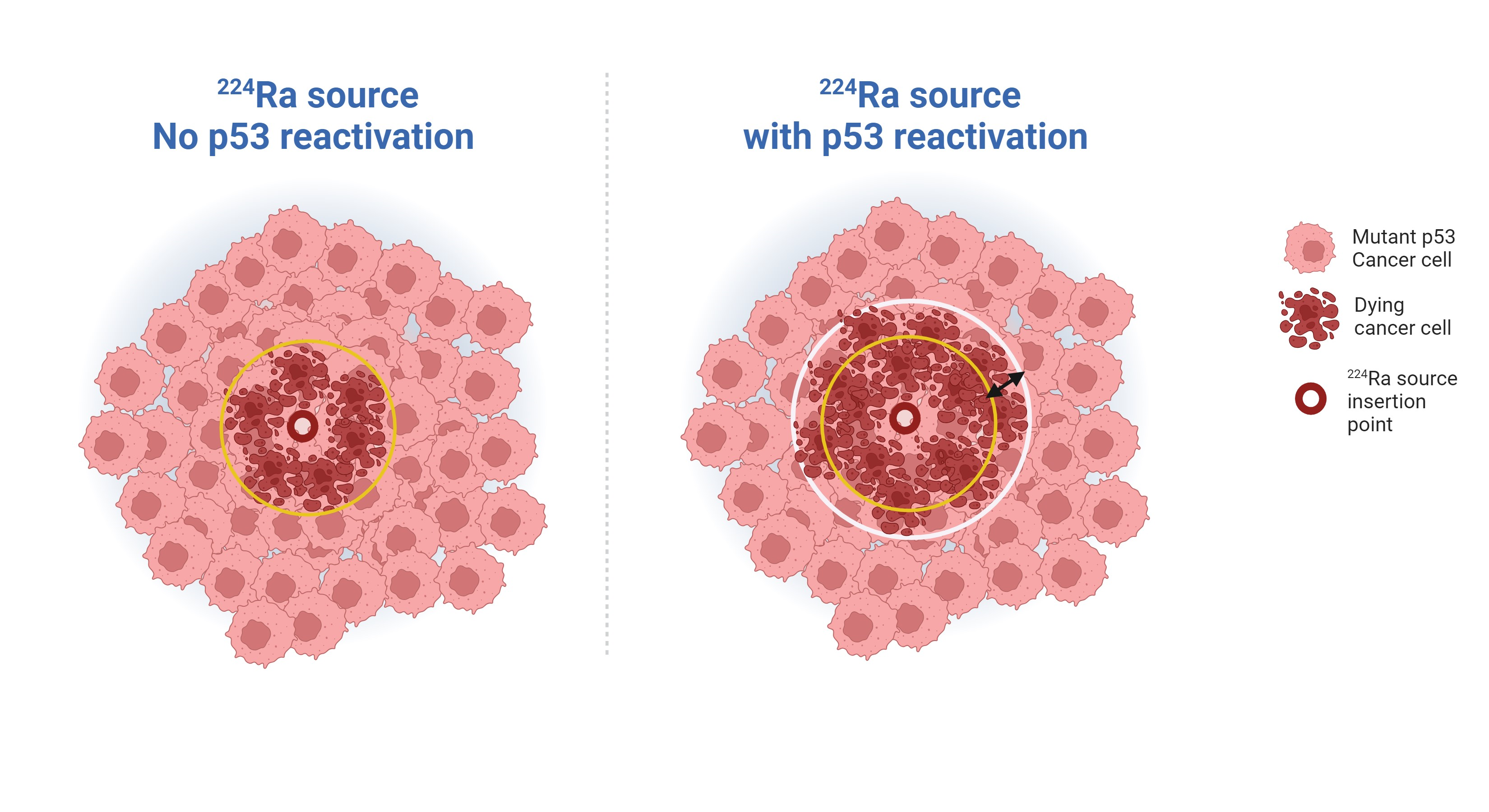

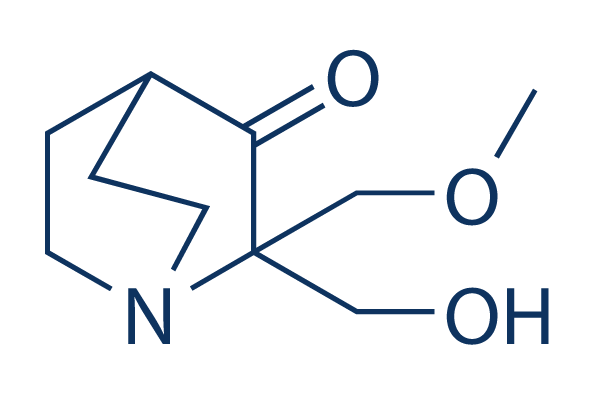


APR-246

**Supp. Figure 6, Graphical representation.** When combined with 224Ra source radiotherapy, the reactivation of the WT form of p53 in tumors harboring GOF mutant p53, will increase the region cell death. Such enlargement of affected perimeters will result in a more efficient tumor control and may be relevant and beneficial to CRC and PDAC patients in the future.
