# Supplementary figures and images for: APR-246 as a radiosensitization strategy for mutant p53 cancers treated with alpha-particles-based radiotherapy
Source: Cell Death Dis. 2024 Jun 18;15(6):426. doi: 10.1038/s41419-024-06830-3 (PMC11189442; doi:10.1038/s41419-024-06830-3)

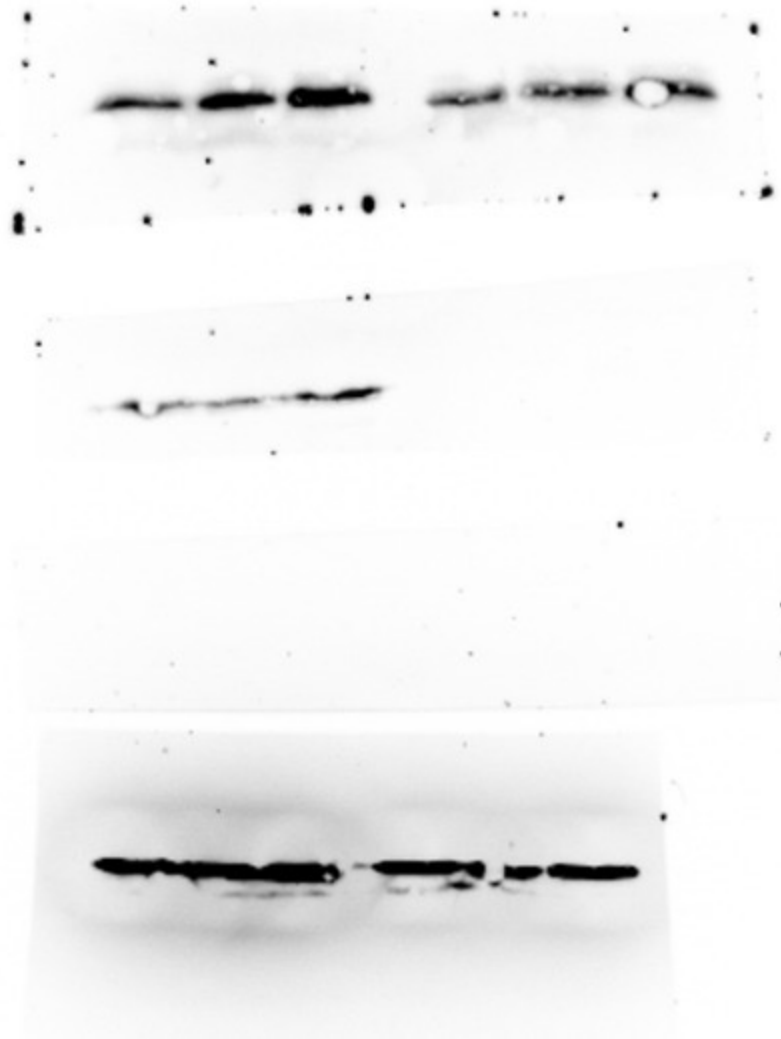

Figure 3G

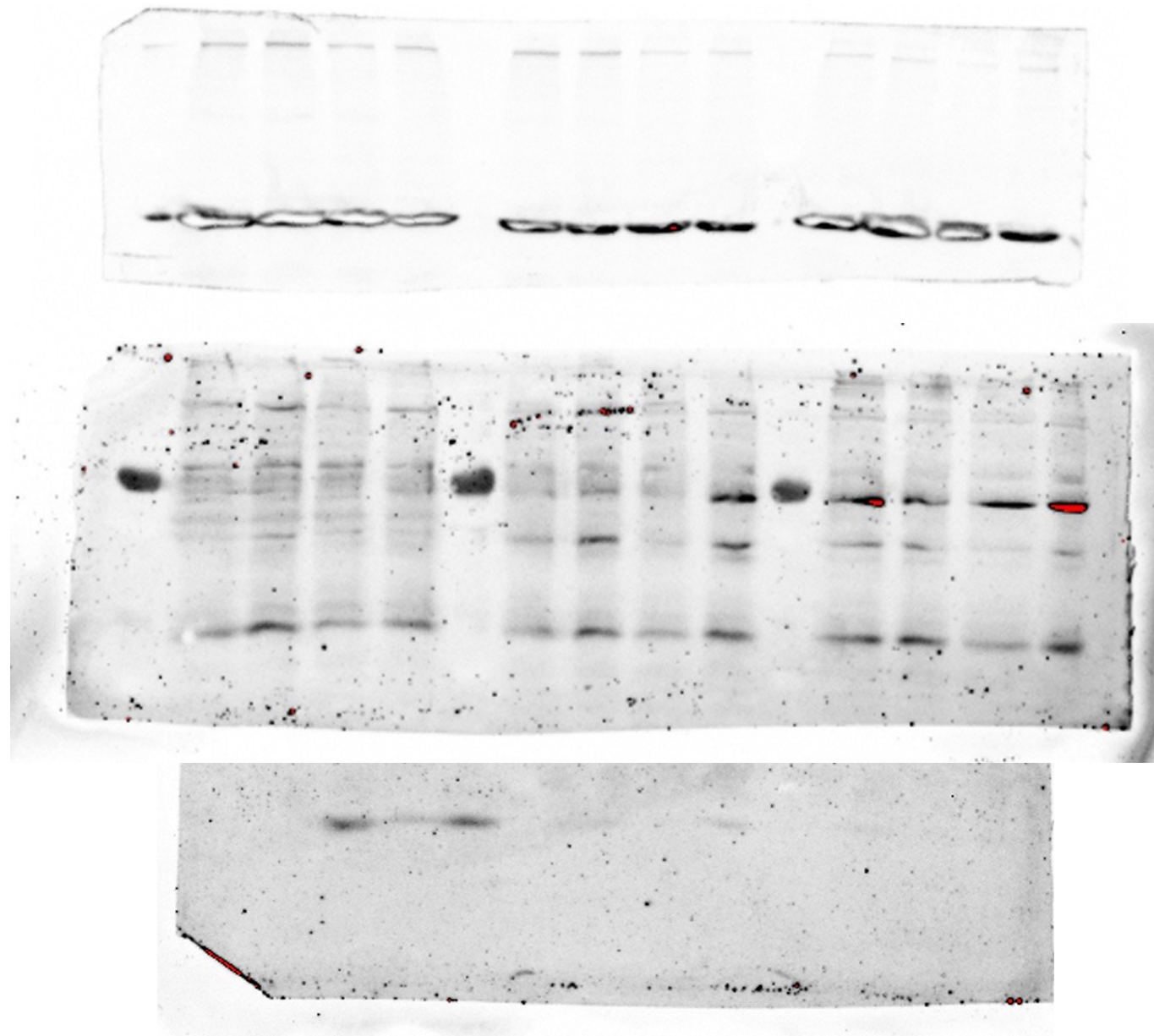

Supp. Figure 3A

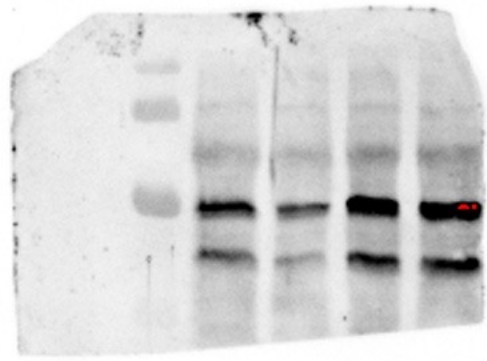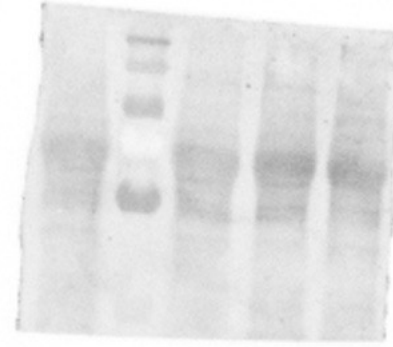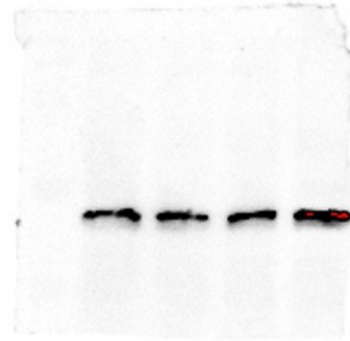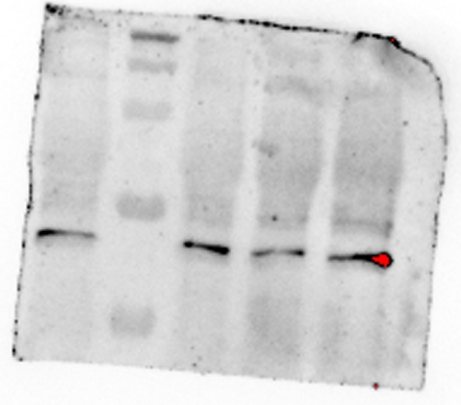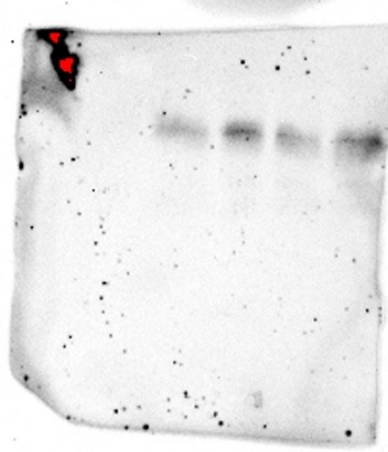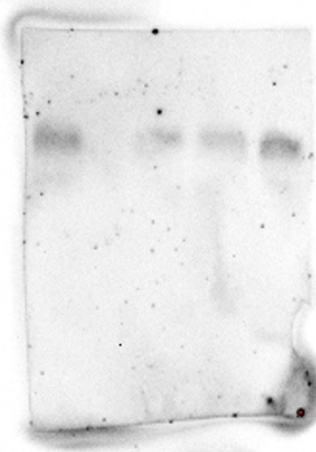

Supp. Figure 3D

Supplement: Supplementary file 2 — Blots [file 41419_2024_6830_MOESM2_ESM.pdf]
